# The molecular and phenotypic makeup of fetal human skin T lymphocytes

**DOI:** 10.1242/dev.199781

**Published:** 2021-10-26

**Authors:** René Reitermaier, Tanya Ayub, Julia Staller, Philip Kienzl, Nikolaus Fortelny, Pablo Augusto Vieyra-Garcia, Christof Worda, Christian Fiala, Clement Staud, Wolfgang Eppel, Anke Scharrer, Thomas Krausgruber, Adelheid Elbe-Bürger

**Affiliations:** 1 Department of Dermatology, Medical University of Vienna, Vienna 1090, Austria; 2 Department of Biosciences, University of Salzburg, Salzburg 5020, Austria; 3 Department of Dermatology, Medical University of Graz, Graz 8036, Austria; 4 Department of Obstetrics & Gynecology, Medical University of Vienna, Vienna 1090, Austria; 5 Gynmed Clinic, Vienna 1150, Austria; 6 Department of Women's and Children's Health, Division of Obstetrics and Gynaecology, Karolinska Institute and Karolinska University Hospital, Stockholm 171 77, Sweden; 7 Department of Surgery, Division of Plastic and Reconstructive Surgery, Medical University of Vienna, Vienna 1090, Austria; 8 Department of Pathology, Medical University of Vienna, Vienna 1090, Austria; 9 CeMM Research Center for Molecular Medicine of the Austrian Academy of Sciences, Vienna, Austria

**Keywords:** T cells, TCR, Skin, Human, Fetal, Single-cell RNA-sequencing, Flow cytometry, Confocal microscopy

## Abstract

The adult human skin contains a vast number of T cells that are essential for skin homeostasis and pathogen defense. T cells are first observed in the skin at the early stages of gestation; however, our understanding of their contribution to early immunity has been limited by their low abundance and lack of comprehensive methodologies for their assessment. Here, we describe a new workflow for isolating and expanding significant amounts of T cells from fetal human skin. Using multiparametric flow cytometry and *in situ* immunofluorescence, we found a large population with a naive phenotype and small populations with a memory and regulatory phenotype. Their molecular state was characterized using single-cell transcriptomics and TCR repertoire profiling. Importantly, culture of total fetal skin biopsies facilitated T cell expansion without a substantial impact on their phenotype, a major prerequisite for subsequent functional assays. Collectively, our experimental approaches and data advance the understanding of fetal skin immunity and potential use in future therapeutic interventions.

## Abstract (precis)

**Summary:** This study provides versatile tools for the isolation, phenotyping and expansion of human fetal skin T cells, enabling the study of their complexity and heterogeneity allowing new research in skin development and immunity.

## INTRODUCTION

The immune system constantly surveys the microenvironment and discriminates between harmless and potentially harmful components – a multifaceted task at barrier surfaces, such as the skin, that are constantly exposed to exogenous stimuli. There, a complex network of cellular and molecular pathways is employed, which allows the immune system to respond quickly and efficiently to harmful stimuli, while largely ignoring innocuous substances. Regulation of both innate and adaptive skin immunity is essential in preserving host integrity, thereby preventing inappropriate immune activation and pathology. T cells, as key actors of the adaptive immune system, are commonly identified by CD3 expression, and detect antigens through heterodimeric T cell receptors (TCRs) composed of either α and β or γ and δ chains (Morath and Schamel, 2020). Work over the last two decades has highlighted their importance in adult human skin, which harbors twice as many T cells as are seen in the circulation (Clark et al., 2006b). Multiple T cell subsets are involved in the defense against pathogens and tumors, and play a role in tissue homeostasis (e.g. hair follicle cycling, wound repair), but they also can cause inflammation and autoimmune diseases (Cruz et al., 2018; Ho and Kupper, 2019).

The developing conceptus is, in principle, protected from pathogens by the uterine barrier and maternal-derived antibodies while establishing a functioning immune network. Skin development is driven by a mutual inductive mechanism between ectoderm (epidermis) and mesoderm (dermis) (Carlson, 2012). The two skin compartments develop over several gestational periods (Ersch and Stallmach, 1999; Coolen et al., 2010). In the first weeks of gestation, the epidermis changes from a single layer of cells into a bilayered epidermis comprising basal cells and an embryonic and fetal-specific periderm (Holbrook and Odland, 1975). Incompletely keratinized cells are replaced by keratinocytes that differentiate while stratifying to form the fully functional epidermis, including the development of skin appendages and a functional stratum corneum between 15-24 weeks estimated gestational age (EGA). The initially dense cellular dermis is followed by augmented production of extracellular matrix components such as collagen fibers, which are detectable at 12 weeks of EGA, with distinguishable papillary and reticular dermis after 15 weeks of EGA (Carlson, 2012; Coolen et al., 2010). The first vessels in the dermis are visible from 11-13 weeks of EGA. The phenotype of cutaneous lymphatic and blood vasculature entirely develops during the second trimester and accumulation of subcutaneous fat begins (Coolen et al., 2010; Schuster et al., 2015; Smith et al., 1986; Colwell et al., 2003). In parallel to the structural changes during the *in utero* development, a diverse range of precursors and immune cells including T lymphocytes seed the skin (Schuster et al., 2009, 2012, 2014) and might be involved in tissue generation and regeneration (Botting and Haniffa, 2020).

In contrast to the well-studied T cells in adult human skin, extensive studies about the establishment of the T cell network in developing fetal human skin have been hampered by a limited amount of and access to fetal tissue, low absolute numbers of T cell per sample and a lack of dissociation methods that liberate viable cells with high yield but preserve T cell markers (Clark et al., 2006b; Schuster et al., 2012; Di Nuzzo et al., 2009; Sanchez Rodriguez et al., 2014). During recent developments in tridimensional visualization and analysis of early human development, high resolution single-cell methods [single-cell RNA-sequencing (scRNA-seq), mass cytometry (cytometry by time-of-flight; CyTOF)], imaging technologies (*in situ* transcriptomics) and computational methods including machine learning algorithms have changed biomedical research. They have facilitated the characterization of immune cell types, generation of diversity in antigen-specific recognition (e.g. TCR repertoire) and tissue compartmentalization immunity across age groups to generate a comprehensive atlas of the human immune system (Park et al., 2020; Mukhopadhyay, 2021; McGovern et al., 2017; Belle et al., 2017; Han et al., 2020) Nonetheless, the precise roles of immune cells such as T cells during skin development remains elusive. Using combinations of traditional and modern laboratory techniques, we carried out investigations on the origin, function and transcriptional profile of fetal skin T cells. Double-positive (DP) αβγδ T cells and single-positive (SP) αβ T cells with a naive phenotype were the predominant population followed by discrete subsets of SP γδ T cells, memory and regulatory T (T_REG_) cells (Reitermaier et al., 2021; Dhariwala et al., 2020).

Here, we provide versatile tools for the isolation and expansion of human fetal skin T cells, which enabled studying their complexity and heterogeneity using single-cell transcriptomics, TCR repertoire profiling, multiparametric flow cytometry and *in situ* immunofluorescence analyses. We thus layout possible future directions for advancing the understanding of skin immunity in early life.

## RESULTS

### Comparison of strategies for a successful isolation of rare fetal skin T cells

**Fig. 1.**
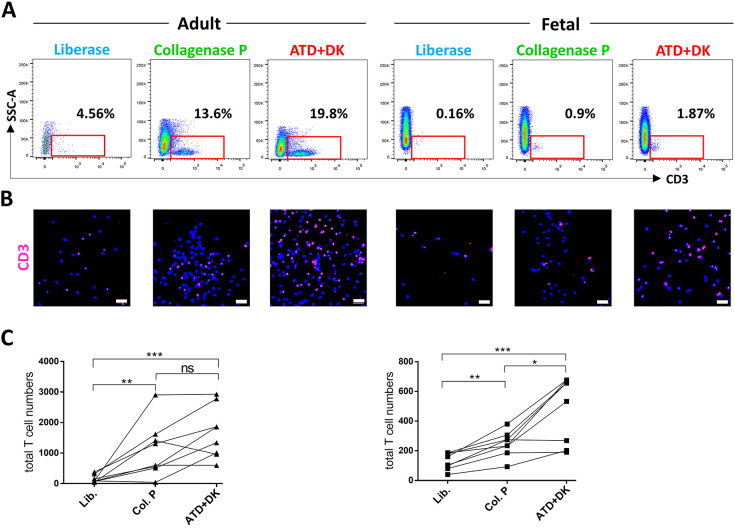
**Comparative T cell isolation procedures.** (A) Representative dot blots demonstrating lymphocytes in adult (30-50 years, *n*=8) and fetal (18-22 weeks EGA, *n*=5) skin gated for CD3 expression upon indicated isolation procedures. ATD, automatic tissue dissociator; DK, dissociation kit. (B) Representative confocal microscope image of adult and fetal skin cells, isolated as in A, on adhesion slides stained for CD3 (red) and DAPI (blue) and analysis. Scale bars: 50 µm. (C) Graphs (adult, left; fetal, right) showing statistical analyses of CD3^+^ T cell frequencies upon denoted isolation procedures for individual donors. Lines connect one individual. Unpaired, two-tailed Student's *t*-test. *P≤0.05, **P≤0.01, ***P≤0.005. ns, not significant.

The isolation of viable T cells from small fetal skin biopsies in sufficient numbers was a challenging first step. T cell yields were comparatively assessed from late second-trimester fetal and adult skin biopsies using established as well as novel isolation techniques and subsequent flow cytometry and confocal microscopy analyses (Fig. 1A,B). As it was technically impossible to efficiently separate epidermis from dermis in fetal skin, unseparated skin, regardless of age, was taken in all experiments. Liberase, containing collagenase I and II, yielded low T cell numbers in both adult (151±44.9 T cells/three biopsies; mean±s.e.m.) and fetal (130±19.9 T cells/three biopsies) skin. Collagenase P enzymatic treatment, an isolation method for adult skin T cells (Salimi et al., 2016), produced significantly more T cells from both adult (1119±318.5 T cells/three biopsies) and fetal (247±30 T cells/three biopsies) skin compared with liberase. The most efficient T cell isolation was obtained with a commercially available automated tissue dissociator (ATD; gentleMACS) and a tissue dissociation kit (DK; Miltenyi Biotec) (adult and fetal: 1666±301.3 and 483±78.9 T cells/three biopsies, respectively) (Fig. 1C). In addition, with this method it was possible to repeatedly isolate low but significant numbers of T cell subsets from fetal skin. Accordingly, all the following experiments were performed using the combination of ATD and DK T cell isolation strategy. Of note, depending on the age and size of the fetus, as many biopsies of each donor were pooled (8-12) as feasible.

### Fetal skin cell types captured by scRNA-seq

**Fig. 2.**
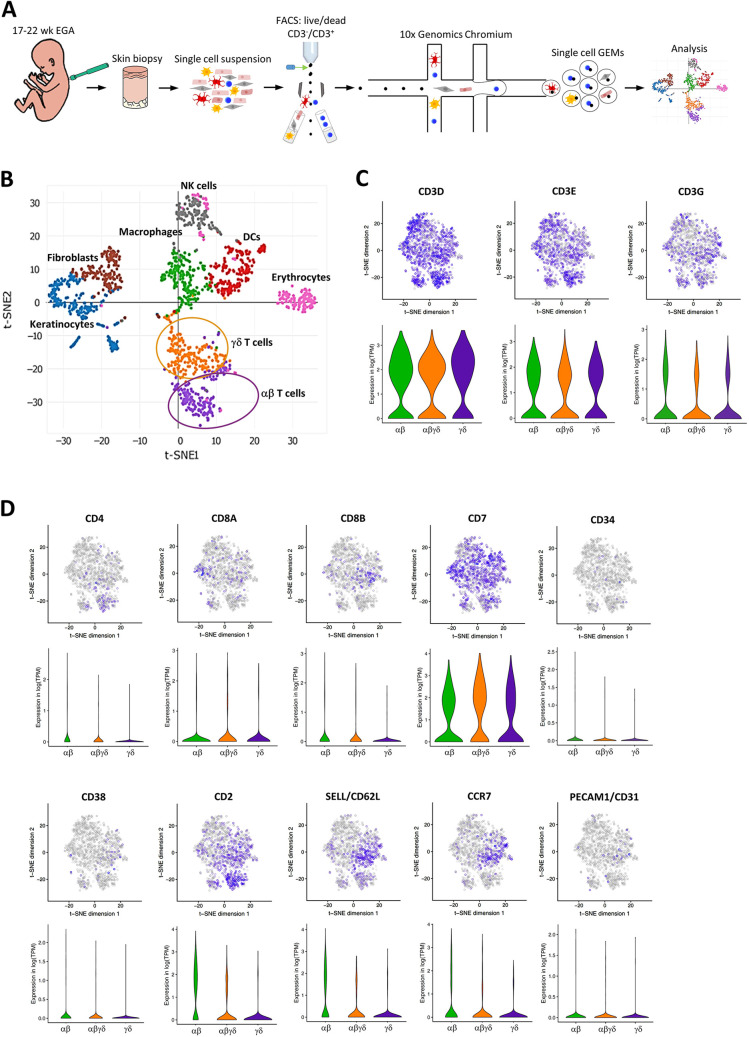
**scRNA-seq map of human fetal skin cells.** (A) Outline of the workflow for single-cell transcriptome profiling of fetal skin biopsies, including dissociation, flow cytometric cell sorting and droplet-based scRNA-seq of cells. GEMs, gel beads-in-emulsion. (B) t-SNE clustering showing cell transcriptomes of two donors (20 and 22 weeks EGA). Each dot represents one cell (1506 in total). Cells are colored and grouped according to their transcription profile. (C,D) The intensity of purple color denotes the normalized level of CD3 subunit genes and hematopoietic as well as T cell precursor gene expression on t-SNE plots of three donors (17, 21 and 22 weeks EGA). Each point represents one cell. Violin plots showing the expression level and distribution of indicated genes.

Sort-purified CD3^+^ T cells were mixed with total fetal skin cells of the same donors for scRNA-seq (10x Genomics) (Fig. 2A) as described in detail previously (Reitermaier et al., 2021). Cell clustering using t-distributed stochastic neighbor embedding (t-SNE) enabled the distinction of major skin cell types including T cells. In total, 1,506 cells with an average of 1,260 unique genes per cell were successfully profiled and analyzed per cell. We detected 19,053 genes using a sequencing saturation of 88%. Based on their transcription level, 400 T cells [further classified according to their TCR expression profile into 184 SP αβ and 216 γδ T cells (100 thereof were DP αβγδ T cells and 116 SP γδ T cells)], 251 keratinocytes, 168 fibroblasts, 138 NK cells, 201 macrophages, 197 DCs and 151 erythrocytes were identified (Fig. 2B). Examination of the top cluster-specific genes in SP αβ and γδ T cells as well as DP αβγδ T cells, a recently described population (Reitermaier et al., 2021), revealed a differential distribution of CD3 subunit genes [*CD3D* (> 90%), *CD3E* and *CD3G*], *CD4*, *CD8A* and *CD8B* genes as well as genes characteristic for hematopoietic stem cells (*CD7*, *CD34*, and *CD38*), T cell precursors and naive T cells [*CD2*, *CD62L* (also known as *SELL*), and *CCR7*], and recent thymic emigrants (*CD31*, also known as *PECAM1*) (Fig. 2C,D).

### Diving into the TCR repertoire in fetal skin T cells

**Fig. 3.**
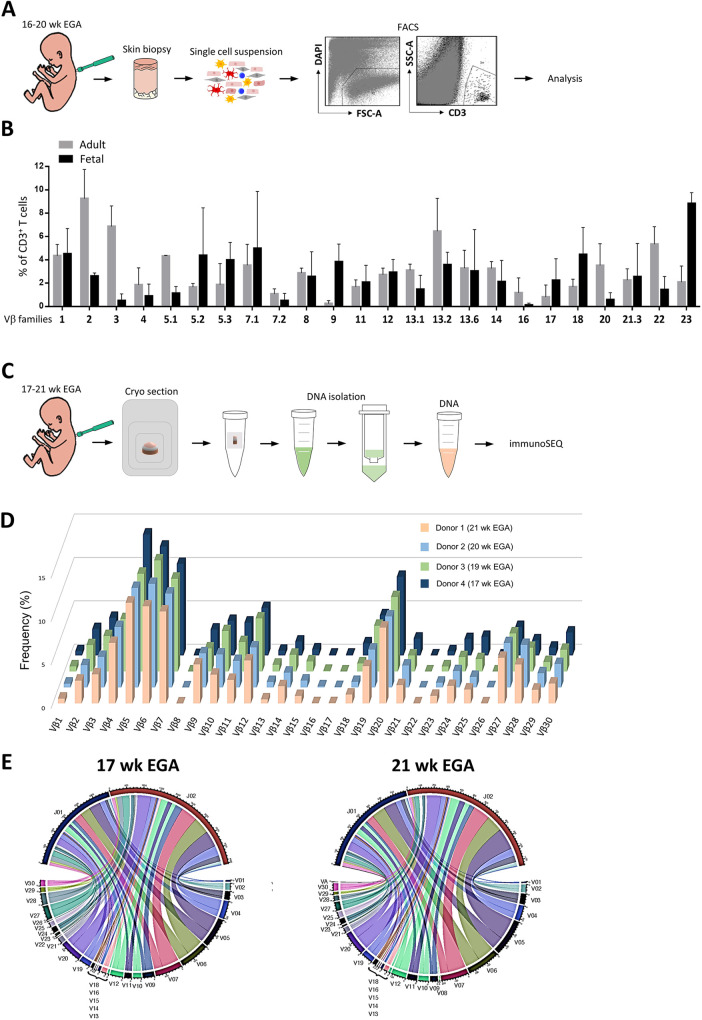
**Distribution of the TCR Vβ repertoire in fetal skin T cells.** (A) Workflow for the evaluation of the TCR Vβ family in fetal skin T cells using flow cytometry analyses. (B) Clonogram (mean±s.e.m.) representing the TCR Vβ repertoire divergence in T cells of fetal (16-20 weeks EGA; *n*=3) and, for comparison, adult (30-50 years; *n*=3) skin cell suspensions. Each value of the individual Vβ family is shown as percentage of total CD3^+^ T cells. (C) High throughput TCR Vβ CDR3 sequencing workflow on DNA obtained from frozen total fetal skin biopsies using immunoSEQ. (D) Frequency distribution of T cell clones according to their constituent Vβ family member. (E) Representative circos plots showing rearrangements of the Vβ and Jβ segments of V (variable), D (diversity) and J (joining)-containing reads.

Several techniques have been developed which have enabled the study of the TCR repertoire (Rosati et al., 2017). The main molecular determinant of T cells, ensuring antigen specificity and strength of the immune response, is the variable (V) region of the TCR. In a first setup, we used antibodies and flow cytometry analyses to study the TCR Vβ family repertoire in fetal skin cell suspensions that were processed alongside adult skin controls (Fig. 3A). Percentages corresponding to the 24 Vβ-specific families of CD3^+^ T cells were plotted as a clonogram. Strikingly, an even distribution of the Vβ family repertoire was observed in fetal skin T cells and was essentially comparable with adult T cells (Fig. 3B).

To analyze the TCR repertoire at a deeper and finer level, high-throughput sequencing was performed. In particular, the frequency of clonal cells by sequencing the complementary-determining region 3 (CDR3) region of the TCR Vβ chain was assessed. Genomic DNA of frozen fetal skin biopsies was sequenced using immunoSEQ technology (Fig. 3C). Each individual TCR V CDR3 profile is depicted as a function of the CDR3 length. A Gaussian CDR3 length distribution pattern was observed for all donors (Fig. S1A). The calculation of the relative frequency for each clone revealed a similar profile of all Vβ families, with slight diversities in each donor (Fig. 3D). As determined by circos plots, no biased usage of TCR Vβ and TCR joining (J) β segments within four different fetal skin donors (17-21 weeks EGA) was observed, implying that the recombination in fetal skin T cells is well-diversified in this gestational age group (Fig. 3E; Fig. S1B).

### Most, but not all, fetal skin T cells display a naive phenotype

**Fig. 4.**
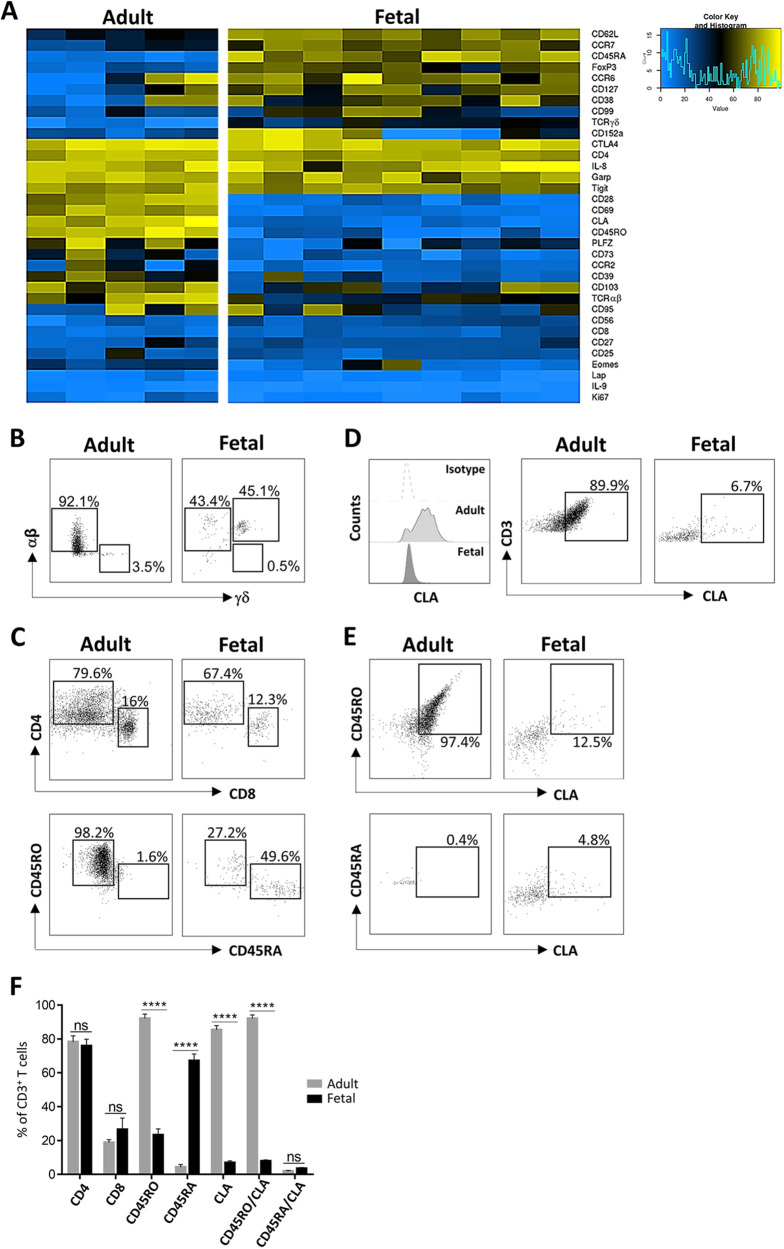
**T cells in fetal skin have a largely naive and proliferative phenotype.** (A) Bi-clustering heat map exhibiting a T cell surface marker expression profile of freshly isolated total T cells of fetal (16-22 weeks EGA; *n*=9) and, for comparison, adult (30-50 years; *n*=5) human skin as analyzed by flow cytometry. Color scheme is based on marker expression ranging from 0% (blue) to 100% (yellow). (B,C) Representative contour plots illustrating freshly isolated CD3^+^ skin T cells expressing indicated markers in adult [44 years (B), 32 years (C)] and fetal [16 weeks EGA (B), 18 weeks EGA (C)] skin as analyzed by flow cytometry. (D) CLA expression on CD3^+^ T cells was analyzed by flow cytometry according to the isotype control in adult (36 years) and fetal (18 weeks EGA) skin. (E) Representative contour plots showing expression of CLA on memory and naïve (38 years versus 18 weeks EGA) T cell subsets in adult and fetal skin. (F) Bar graphs (mean±s.e.m.) revealing expression of indicated markers on CD3^+^ T cells in adult and fetal skin as analyzed by flow cytometry (*n*=8). Unpaired, two-tailed Student's *t-*test. ****P≤0.0005. ns, not significant.

T cell subsets – including naive, effector and memory – can be distinguished from one another via a combination of markers (CD4, CD8, CD45RA, CD45RO, CD62L, CCR7, etc.). A bi-dimensional heat map denotes high and low expression of selected surface markers on total CD3^+^ T cells and demonstrates a distinct marker expression profile of fetal and adult skin T cells as analyzed by flow cytometry (Fig. 4A). Confirming our previously reported results (Reitermaier et al., 2021), αβ and γδ T cell subsets in adult and fetal skin are differentially distributed (Fig. 4B). Furthermore, only fetal skin and intestine contains a T cell subset with a unique TCR co-expressing αβ and γδ chains (DP αβγδ T cells; Fig. 4B) (Reitermaier et al., 2021). Cell surface glycoproteins CD4 and CD8 serve as co-receptors with the TCR primarily for the interaction with the major histocompatibility complex (MHC) class II (MHC II) loaded with peptides derived from cytosolic proteins and MHC I with extracellular protein peptides, respectively. The percentage and expression level of CD4^+^ T cells was consistently, though not significantly, lower in fetal (75.8%±4.1%; *n*=8) compared with adult (78.3%±3.6%; *n*=8) skin. In contrast, repeatedly but not significantly more CD8^+^ T cells were present in fetal skin (26.5%±6.8%), with similar CD8 expression levels when compared with the CD8^+^ T cell population in adult skin (18.8%±1.7%) (Fig. 4A,C,F). The majority of T cells in adult skin had a memory phenotype (92.1%±2.6%) confirming reported results (Clark et al., 2006b), whereas only one-quarter of fetal skin T cells expressed CD45RO (23.3%±3.6%), thus extending results that were assessed by immunohistochemistry and recent (mass) cytometry analyses (Di Nuzzo et al., 2009; Reitermaier et al., 2021; Dhariwala et al., 2020) (Fig. 4A,C,F). Conversely, most fetal T cells were naive (67.1%±4.0%) compared with a small population of CD45RA^+^ T cells in adult skin (4.3%±1.6%) (Fig. 4A,C,F). The majority of resident CD45RO^+^ T cells in adult skin co-expressed the cutaneous lymphocyte antigen (CLA) (92.06%±2.1%), a skin lymphocyte homing receptor, whereas only a few CD45RA^+^ T cells (1.88%±0.516) were positive for this marker (Fig. 4A,D-F), corroborating previously reported findings in adults (Clark et al., 2006b). Small but distinct subsets of both CD45RO^+^CLA^+^ (7.9%±0.8%) and CD45RA^+^CLA^+^ (3.5±0.7%) T cells were identified in fetal skin (Fig. 4A,D-F). Of note, the minute population of CLA^+^ T cells in fetal skin precluded a more precise phenotyping. Further, we observed minor variabilities in the percentage of CLA^+^ cells between different fetal donors (Fig. 4D,E) even though they were of the same age (18 week EGA). We had similar findings in adult donors (36 years versus 38 years) (Fig. 4D,E).

### Fetal skin comprises naive and memory T cell subsets *in situ*

**Fig. 5.**
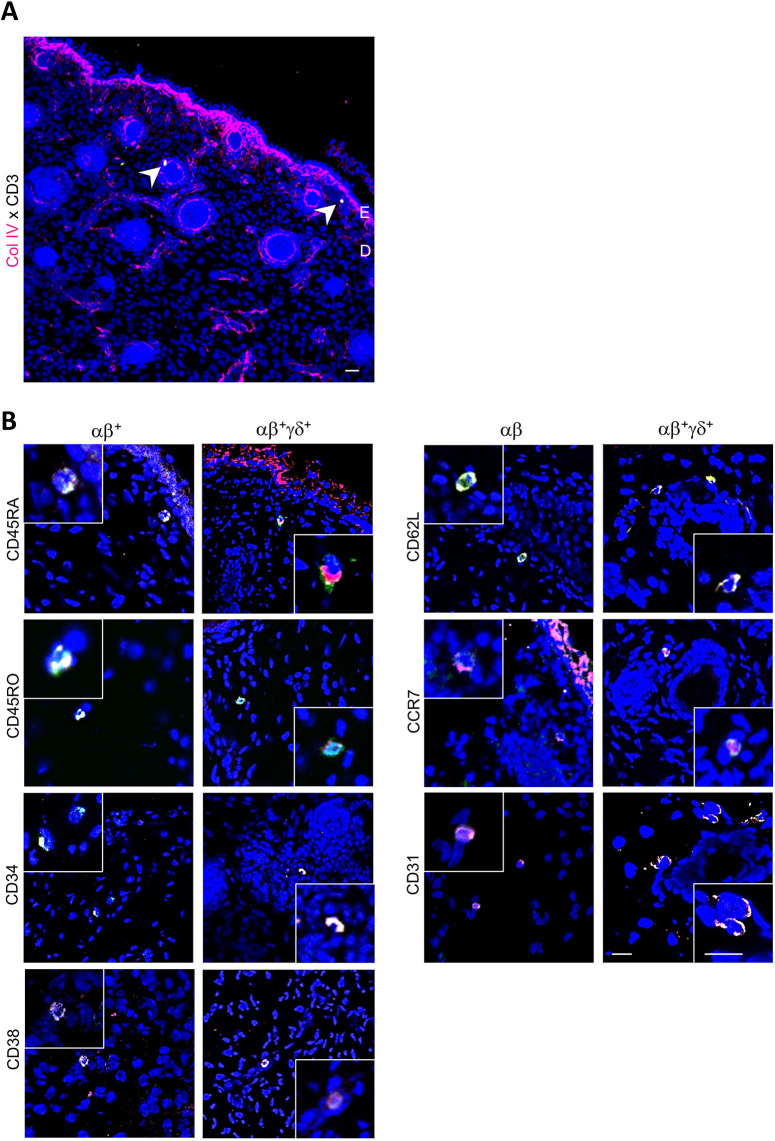
**Distinct T cell subsets are present in fetal human skin *in situ*.** (A) CD3^+^ T cells (arrowheads) can exclusively be found in the dermis in fetal skin, often in the vicinity of hair follicles (15-20 weeks EGA; *n*=12). Nuclear counterstain was performed with DAPI (blue) and collagen type IV (magenta) visualizes the dermo-epidermal junction. (B) Immunofluorescence quadruple labeling and DAPI counterstaining for the markers indicated was performed on fetal skin cryostat sections and assessed by confocal laser microscopy. Images for each marker combination are representative from at least three different donors with similar results (17-22 weeks EGA; *n*=30). Scale bars: 10 µm (A); 20 µm (B). D, dermis; E, epidermis.

We next sought to discover whether T cell subsets, as shown by flow cytometry (Fig. 4), can also be identified in fetal skin *in situ*. Of note, irregular CD3^+^ T cells were observed in second trimester fetal skin exclusively in the dermis and, in line with a previous report (Dhariwala et al., 2020), sometimes in the vicinity of hair follicles (Fig. 5A). Quadruple immunofluorescence staining of fetal skin sections revealed SP αβ and DP αβγδ T cells expressing CD45RA rather than CD45RO (Fig. 5B; Fig. S2A), markers for hematopoietic stem cells (CD34, CD38; Fig. 5B; Fig. S2B), naive T cells (CD62L, CCR7; Fig. 5B; Fig. S2C), as well as recent thymic emigrants (CD31; Fig. 5B; Fig. S2D). SP γδ T cells (Reitermaier et al., 2021) as well as CD1a-expressing cells were undetectable in the samples analyzed (Fig. S2D). As T cells *in situ* are irregular and rare, their quantification was unfeasible.

### Fetal skin T cells can be propagated *ex vivo* without substantial impact on their phenotype

**Fig. 6.**
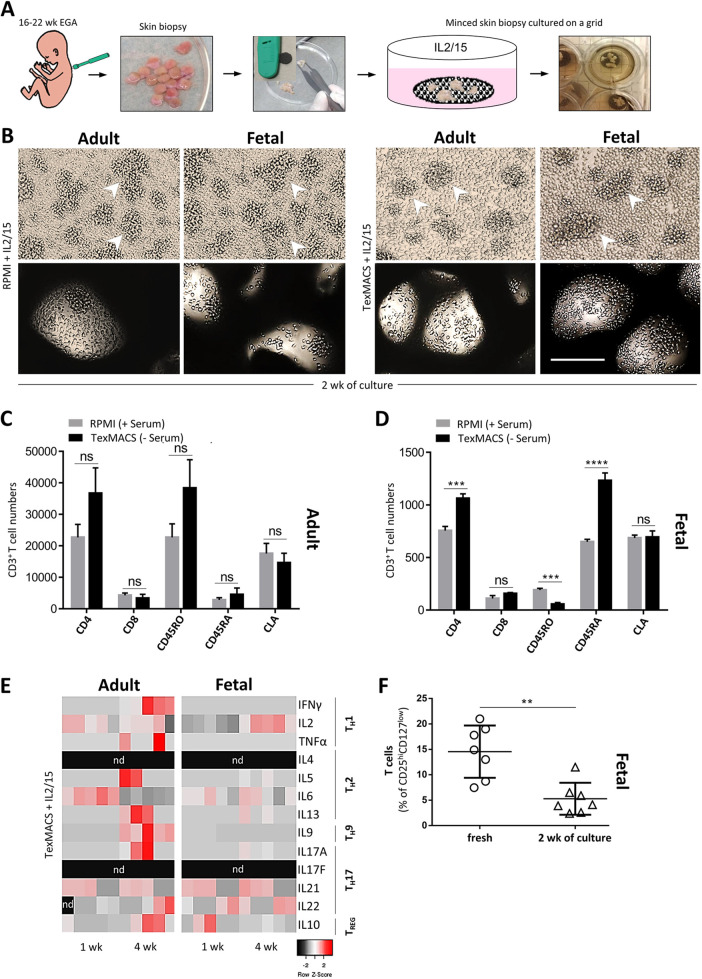
**T cells can be expanded from fetal skin *in vitro*.** (A) *Ex vivo* skin T cell expansion workflow. (B) Representative T cell clusters (arrowheads), indicating proliferation, are visible upon culturing of fetal (*n*=5) and adult (*n*=8) skin specimens on collagen-treated grids in the presence of IL2/15 and either RPMI or TexMACS medium after 2 weeks. Upper panels, nearby grid; lower panels, grid area. Scale bar: 200 μm. (C,D) Bar graphs (mean±s.e.m.) showing numbers of expanded CD3^+^ T cells of fetal (*n*=5) and adult (*n*=8) skin specimens with indicated culture conditions upon 2 weeks and flow cytometry analyses. (E) Heat map of signature cytokines for each T cell subset identified in supernatants of fetal and adult skin specimen cultures (TexMACS and IL2/15) and measured with a cytometric bead assay at 1 and 4 weeks (*n*=5/age group). The color bar demonstrates the *z*-score. (F) A significant decrease of T_REG_ cells in fetal skin cell cultures with TexMACS medium and IL2/15 was observed after 2 weeks (*n*=7). Unpaired, two-tailed Student's *t-*test. **P≤0.006, ***P≤0.0004, ****P≤0.0001. nd, not detected; ns, not significant.

Even though isolation and sorting of significant viable T cell numbers from freshly isolated fetal skin is possible, extensive studies remain challenging. Therefore, we aimed to set up a skin culture system suitable to expand viable T cells from fetal skin. To this end, we explored an *ex vivo* culture system previously used to expand T cells from both healthy and diseased adult human skin (Clark et al., 2006a), to investigate its applicability for fetal skin. In this setup, fresh fetal skin biopsies alongside adult skin controls were cut into small pieces and cultured on collagen-coated grids using two different culture media (RPMI plus serum and serum-free TexMACS) (Fig. 6A), in the presence and absence of IL2/15. T cells started to spill from the matrices on day 3 and proliferating clusters with an approximately similar size, both small and large in shape, were visible in cultures with fetal skin and adult control skin after 2 weeks (Fig. 6B, arrowheads). Without cytokines, only single T cells and markedly less proliferation was observed compared with cytokine-containing cultures, regardless of skin age and medium used (Fig. S3A,B). Expanded T cells from adult skin predominantly expressed CD45RO (Fig. 6C; Fig. S3B), whereas the majority of fetal skin T cells were positive for CD45RA (Fig. 6D; Fig. S3B). These data clearly demonstrate that the culture conditions do not facilitate the outgrowth of a particular T cell subset and rather reflect the situation before culture. Of note, in contrast to the small percentage of freshly isolated fetal T cells expressing CLA (Fig. 4), several expanded fetal T cells expressed this marker (Fig. 6D; Fig. S3B). Fibroblasts were regularly visible in cultures with RPMI upon 2-4 weeks, but scarcely in cultures with TexMACS (Fig. S3D). To determine signature cytokines for each T cell subset, supernatants collected at 1 and 4 weeks from fetal and adult skin cultures (TexMACS±IL2/15) were analyzed with a LEGENDplex bead array (Fig. 6E; Fig. S3C). T_H_1 and T_H_2 cytokines were not measurable in supernatants derived from fetal and adult skin explants and TexMACS medium only (Fig. S3C). In contrast, culture of skin biopsies with IL2/15 initiated secretion of T_H_1 and T_H_2-related cytokines but was more pronounced in supernatants from cultures with adult skin, particularly after 4 weeks of culture, whereas it appeared fairly unchanged at both time points in supernatants from fetal skin (Fig. 6E). Cytokines specific for T_H_9, T_H_17 and T_REG_ cells could be observed in supernatants of both adult and fetal skin specimens cultured with or without cytokines after 1 week, but was more prominent in 4 week cultures of adult skin (Fig. 6E; Fig. S3C). Of note, high levels of the suppressor cytokine IL10 were measured in supernatants of fetal skin cultures after 1 week, regardless of whether cytokines were present or not in the culture medium, but these decreased after 4 weeks (Fig. 6E; Fig. S3C). The IL10 decline with culture duration (Fig. 6E) correlated with a decrease of T_REG_ cells (Fig. 6F).

## DISCUSSION

In this study, we used a combination of advanced approaches to further explore the complexity and heterogeneity of T cell subsets in fetal human skin. We describe methods for the isolation and expansion of rare fetal skin T cells. Further, single-cell transcriptomics and TCR repertoire profiling mapped the molecular state of fetal skin T cells, and multiparametric flow cytometry as well as *in situ* immunofluorescence analyses were integrated to validate the molecular-based profile, thus contributing to a better understanding of the particularities of fetal skin immunity.

The isolation of T cells from small fetal skin biopsies in sufficient numbers and with robust and reliable quality was a challenging first step towards studying their nature and function. To address this, we comparatively assessed T cell yields from fetal and adult human skin biopsies using several isolation techniques, identifying a combination of automated and enzymatic dissociation as the most efficient and reproducible procedure. Importantly, this methodology preserved pan surface markers such as CD3, CD4, CD8, CD45RA and CD45RO that are necessary for the phenotypic characterization of T cell subsets and will be also a useful tool for studying skin biopsies with limited patient material in the future.

Recognized for its high sensitivity (Zhao et al., 2018), we have used 10x Genomics for single-cell transcriptome profiling of fetal skin to resolve the cellular heterogeneity. The major cell types described in adult skin (Rojahn et al., 2020), were also identified during early development, although differing in relative abundance. Of note, other groups have identified B cells, precursors and other immune populations in human fetal skin (Botting and Haniffa, 2020; Dhariwala et al., 2020; Popescu et al., 2019), which were absent in our samples most likely due to the fact that the majority of cells were sorted CD3^+^ T cells that were mixed with total skin cells as our primary goal was to characterize T cells. We reported previously that beside conventional SP αβ and γδ T cells, a DP αβγδ fetal skin T cell population is unique to the early fetal period and is absent in the skin at the time of birth and in healthy adults (Reitermaier et al., 2021). All three T cell subsets showed gene expression for CD3 subunits, classical hematopoietic stem cells and T cell precursors. Multiparametric flow cytometry and *in situ* immunofluorescence analyses validated and expanded the transcriptome-based profile, and revealed that the majority of fetal skin T cells were positive for CD4, expressed markers that are characteristic for naive T cells (CD45RA, CD62L, CCR7) as well as hematopoietic stem cells (CD34 and CD38). Observations in sheep suggested a pathway of recirculating naive T cells within fetal skin to establish tolerance to self-antigens (Cahill et al., 1999). In support of this are published data that show CD45RA^+^CD8^+^CD62L^−^CCR7^−^ T cells expressing the skin homing marker CLA in human cord blood lymphocytes (Zippelius et al., 2004). Although we observed a small CD45RA^+^CLA^+^ T cell population in fetal skin (Fig. 4B-D), it remains to be investigated whether they express CD4, CD8, CD62L and/or CCR7. We have previously reported that more than two-thirds of all naive T cells in fetal skin express CD31, indicative for recent thymic emigrants (Reitermaier et al., 2021), which is in line with a recent study reporting on elevated CD31 expression in CD45RO^−^ fetal skin T cells (Dhariwala et al., 2020). Although our observation that DP αβγδ fetal skin T cells expressing CD31 are undetectable in the thymus suggest their extrathymic development (Reitermaier et al., 2021), the derivation of CD31^+^SP αβ and γδ T cells needs to be further explored.

Adult human skin is protected by two discrete populations of resident memory T cells and two distinct populations (CCR7^+^CD62L^+^ and CCR7^+^CD62L^−^) of recirculating skin-tropic (CLA^+^) T cells, each with different functional capacities (Clark et al., 2006b; Watanabe et al., 2015). Strikingly, fetal skin also harbors CLA^+^ memory T cells (Di Nuzzo et al., 2009) (Fig. 4B-D) but, in contrast to adult skin, in the absence of a reported pathology or any major infectious history. Their minute frequency correlates with the observation of low levels of the T cell-attracting chemokine CCL27 (also known as CTACK) (Morales et al., 1999) in fetal compared with adult (Schuster et al., 2012; Mildner et al., 2014) skin precluding an influx of memory T cells, if present, from the circulation (Zhang et al., 2014). Keratinocytes fail to produce CCL27 correlating with its negative staining in the first trimester. Strong specific CCL27 expression was observed only in the stratum corneum towards the end of the second trimester, indicating a differentiation-dependent regulation. These observations implied that seeding of fetal skin with naive T cells occurs independently of CCL27 (Schuster et al., 2012). The structural and functional immaturity of the epidermis in early fetal development may also explain why neither naive nor memory T cells have been identified in this compartment (Schuster et al., 2012; Di Nuzzo et al., 2009; Reitermaier et al., 2021; Dhariwala et al., 2020). Changes in the microenvironment and the keratinocyte differentiation process with increasing CCL27 levels in keratinocytes seem to favor a gradient of T cell influx, as naive and memory T cells were identified not only in the dermis but also in the epidermis after birth (Akgün et al., 2014). Of note, also in adult skin CCR7^+^CD62L^−^CLA^+^ migratory memory T cells were reported to be confined to the dermis and absent from the epidermis (Watanabe et al., 2015).

T cells are defined by their TCR sequences, facilitating the accomplishment of highly specific TCR-dependent antigen recognition. The antigen recognition triggers downstream signaling of T cells – a crucial biological process (Jung and Alt, 2004). The TCR repertoire hence represents a ‘footprint’ of the conditions faced by T cells that dynamically evolves according to the challenges that arise for the immune system. Consequently, profiling the TCR repertoire was of interest in our study. Unexpectedly, a relatively consistent TCR Vβ family repertoire was found, with small variations in the individual Vβ segments in fetal skin T cells and was comparable with T cells in adult skin. As our data with adult skin are in line with those obtained in a previous study, we furthermore demonstrate the robustness and reproducibility of this assay (Clark et al., 2012). Of note, an increase (5.2, 5.3, 7.1, 9, 17, 18 and 23) and a decrease (2, 3, 5.1, 13.1, 13.2, 16, 20 and 22) of particular Vβ families was observed in fetal compared with adult skin. However, further work is needed to understand this distinct expression profile. High-throughput sequencing of the CDR3 region indicated the occurrence of a diverse skin TCR repertoire, and an analogous distribution in fetal skin T cells between four unrelated donors. In addition, assessments of the rearrangement in the same donors showed quite similar TCR Vβ and TCR Jβ combinations. This is in contrast to previous studies reporting a skewed usage of TCR Vβ families during development (Rechavi et al., 2015; Carey et al., 2017). This discrepancy could be explained using different techniques and tissues. Together, our analyses are the first to unravel the TCR repertoire of fetal skin T cells. We are aware that the unknown functional relevance of TCR profiling hinders unbiased interpretation of the biology of T cells. Most recently, a tool (tessa) has been developed enabling mapping of the functional landscape of the TCR repertoires by combining scRNA-seq with TCR sequencing (Zhang et al., 2021). Its application could allow answer a variety of research questions regarding biology of fetal skin T cells in the future.

Given the limitations to obtain sufficient T cell numbers from fetal skin, we aimed to expand T cells from fetal skin *ex vivo*. We embarked on a method established for the expansion of T cells from adult human skin that has greatly facilitated studies of this important population (Clark et al., 2006a). Although both media (RPMI, TexMACS) favored the expansion of fetal skin T cells, we observed significantly better cell yields using TexMACS. Although T cell numbers isolated from different fetal donors varied, the kinetics of T cell expansion was comparable for all donors and, most importantly, maintained their naive phenotype. Of note, about 50% of expanded fetal T cells, irrespective of whether cultured with or without serum, expressed CLA. This is in contrast to the small population expressing this molecule at culture initiation and to a previous report showing that optimal CLA induction is only possible in the absence of serum (Armerding and Kupper, 1999). Our data may suggest an expansion of CLA^+^ T cells rather than its induction due to culture conditions and remains to be further investigated. Together, expanded T cells essentially maintain the properties they have *in utero* and can be used as a model for researchers studying basic biological processes.

Regarding signature cytokines for T cell subsets, we identified a shift towards T_H_1, T_H_2 and T_H_9 subsets in supernatants of adult but not fetal skin cultures. The high IL9 levels after 4 weeks of adult skin culture are either due to higher numbers of T_H_9 cells or, alternatively, due to the proliferation of other IL9-producing T cell subsets such as T_H_2 and/or T_H_17 cells which cannot be distinguished in fetal skin (Schlapbach et al., 2014). Of note, the higher levels of the cytokine IL22 in fetal cultures, irrespective of culture conditions and duration, suggest that SP γδ and/or DP αβγδ T cells may be the cellular source (Reitermaier et al., 2021; Mielke et al., 2013).

Today we know that the immune system is not fundamentally immature in early life, but simply differs from immune responses observed in later life. Recent studies indicate that a predisposition of the fetal immune system toward tolerance is assignable to both lymphocyte intrinsic and dendritic cell-dependent features (McGovern et al., 2017; Mold and McCune, 2012), which are involved to completely avoid or regulate responses to self, maternal or foreign antigens *in utero*. We have reported previously that IL10 concentrations in fetal skin single-cell cultures are 18- to 50-fold higher than in healthy adult skin (Schuster et al., 2009), and suggested that the developing skin represents an immunosuppressive environment. Using another approach by culturing fetal skin pieces in a serum-free medium, high levels of the immunomodulatory cytokine IL10 (data not shown) were measured in supernatants after 1 week, but greatly declined with culture duration. Remarkably, this regression correlated with the decrease of T_REG_ cells, which are an ‘abundant’ population (∼10-20%) within fetal skin T cells (Schuster et al., 2012; Sanchez Rodriguez et al., 2014; Dhariwala et al., 2020). Our results are indicative that T_REG_ cells might represent one of the main producers of suppressor cytokines in fetal skin and thus contribute to the fetal immunosuppressive environment. Suboptimal conditions for their expansion may be one explanation for their disappearance in our cultures. Of note, even though T_REG_ cells in fetal skin are quite similar to their adult counterpart regarding their effector memory profile, modest age-related differences in key markers may indicate distinct functional capacity of these cells by age (Dhariwala et al., 2020). Further, although multiple mechanisms for T_REG_ cell suppression have been shown *in vitro*, it is still unclear whether the same or different mechanisms are used by T_REG_ cells *in vivo* (Shevach, 2009). Addressing this is not trivial and will need sophisticated experiments in the future.

Collectively, we describe a workflow for the isolation, phenotyping and expansion of human fetal skin T cells that will drive new research directions in skin development and immunity, enabling discoveries in the treatment of neonatal skin infection and skin diseases.

## MATERIALS AND METHODS

### Human skin and consent

Specimens of fetal trunk skin (15-22 weeks EGA) were taken after legal termination of pregnancy. Adult skin (25-51 years) was obtained from healthy volunteers after abdominal cosmetic surgery. The study was approved by the local ethics committee of the Medical University of Vienna and conducted in accordance with the Declaration of Helsinki Principles. Women and participants gave their written informed consent. Tissue samples were disinfected with Kodan disinfectant (Schülke & Mayr) and cleaned with phosphate-buffered saline (PBS; Gibco) before cell isolation.

### Preparation procedures for skin single-cell suspensions

To be able to compare cell yields by using different isolation techniques and to avoid technical variance it was mandatory to use the same amount of fetal and adult skin (three punch biopsies at 4 mm diameter; Kai Europe GmbH) from the identical donor. Accordingly, skin biopsies were incubated with 1.2 U ml^−1^ dispase II (Roche Diagnostics) in PBS (overnight, 4°C). The next day, 0.53 U ml^−1^ liberase 3 (Roche Diagnostics) was added and shaken (90 min, 37°C). In parallel, skin biopsies were incubated with 1.6 U ml^−1^ collagenase P in RPMI 1640 medium (Invitrogen). After incubation (overnight, 37°C, 5% CO_2_), 10 μg/ml DNase I (Roche Diagnostics) was added to collagenase P-containing tubes. In addition to these two separation methods, a human skin DK (Miltenyi Biotec, whole skin dissociation kit, human) in combination with an ATD (gentleMACS Octo dissociator, Miltenyi Biotec) was used. Briefly, punch biopsies (three punch biopsies at 4 mm diameter) were incubated with appropriate enzymes as described in the manufacturer's instructions overnight at 37°C. Subsequently, the dissociation kit samples were put onto gentleMACS for mechanical treatment. The ‘h_skin_01’ preinstalled program was used. Cell clumps and tissue debris were removed with a 70 μm nylon cell strainer after all preparation procedures. Remaining cells were washed with PBS and cells analyzed on adhesion slides by confocal microscopy and flow cytometry.

### Adhesion slides

Freshly isolated single-cell suspensions from fetal and adult skin biopsies were transferred onto adhesion slides (Marienfeld), applying 50 µl (20,000 cells/well) per reaction field and incubated (10 min, room temperature). After washing the slides with PBS, they were fixed in ice-cold acetone (10 min; Merck) and either immediately stained or frozen at −20°C until further processing.

### Flow cytometry and cell sorting

Single-cell suspensions prepared from several fetal and adult human skin biopsies were stained with labeled monoclonal and recombinant antibodies (Table S1) and appropriate isotype controls. Gating strategy included discrimination of doublets and dead cells with 4′,6-diamidino-2-phenylindole (DAPI; Sigma-Aldrich). Analyses were performed on Aria II/III and fluorescence-activated cell sorting (FACS) Verse (BD Biosciences) and data evaluated with FlowJo software (Tree Star; V_10). Bi-dimensional heat map analysis of selected marker expression was performed using the R software (http://www.r-project.org/). In certain experiments, viable CD3^+^ T cells were sorted (up to 99% purity) from freshly digested tissue cell suspensions on Aria II/III and subjected to scRNA-seq processing immediately after sorting as indicated.

### Droplet-based scRNA-seq

For TCR and transcriptional profiling of fetal human skin cells, the Chromium Controller (10x Genomics) was used according to the manufacturer's protocol and analysis carried out as previously described (Reitermaier et al., 2021). Viable, sorted CD3^+^ T cells (up to 5,000 cells/fetus) were mixed with sort-purified total skin cells (ratio up to 1:1) to get a more complete resolution of human skin development. Detailed description of scRNA-accomplishment and analysis have been described in detail recently (Reitermaier et al., 2021). However, results presented herein address other aspects of analysis. All data are listed in GEO under accession number GSE156972.

### TCR Vβ evaluation

Single-cell suspensions prepared from fetal and adult human skin biopsies were stained with the allophycocyanin (APC)-labeled monoclonal antibody (mAb) anti-CD3 (Invitrogen) (Table S1). Subsequent Vβ staining, including appropriate isotype controls, was performed with the IOTest beta Mark Vβ Repertoire Kit (Beckman Coulter) according to the manufacturer's instructions and analyzed with a FACS Verse. Dead cells were excluded with DAPI and data analyzed with FlowJo software.

### High-throughput TCR sequencing

DNA was isolated from frozen, in optimal cutting temperature (O.C.T.; Tokio) compound-embedded fetal skin samples with the DNA Mini Kit 50 (Qiagen), and used to amplify the CDR3 of the TCR Vβ chain for sequencing analysis by ImmunoSEQ (Adaptive Biotechnologies) according to the manufacturer's instructions. Circos plots were performed using R software (http://www.r-project.org/).

### Immunofluorescence

We mounted 5 μm fetal skin cryostat sections on capillary gap microscope slides, fixed in ice-cold acetone for 10 min, air dried, and incubated in a humid chamber with antibodies (Table S1; 1:50 dilution; 1 h, 4°C). After washing with PBS, slides were stained with DAPI, washed with PBS, mounted with fluoprep (bioMérieux) and analyzed with a confocal laser scanning microscope (LSM 780; Carl Zeiss) equipped with a highly sensitive 32-channel gallium arsenide phosphide photomultiplier tube area detector (AiryScan; Carl Zeiss) that collects a pinhole-plane image at every scan position. Each detector element functions as a single very small pinhole and enables very light-efficient imaging with improved resolution and signal-to-noise ratio.

### *In vitro* T cell expansion from skin biopsies

We cut 4 mm punch biopsies into small pieces (∼1 mm) and transferred onto three-dimensional collagen-coated cellfoam matrices (grids, 9 mm×1.5 mm; Cytomatrix Pty) (Reitermaier et al., 2021). The charged grids were transferred, in triplicates, into wells of a 24-well plate (Beckton Dickinson Labware Europe) containing either 2 ml RPMI plus 10% fetal calf serum (FCS) or TexMACS medium, a serum-free cell culture medium, developed for the cultivation and expansion of human T cells, with 1% penicillin/streptomycin, and with or without a combination of cytokines (IL2, 100 U/ml; IL15, 10 ng/ml; both obtained from PeproTech). At selected time points cells were harvested, centrifuged and the supernatant aspirated and frozen. Cells were analyzed by flow cytometry. Expansion of T cells has been documented throughout the culture period.

### Analysis of cytokines in culture supernatants

T cell subset-related cytokines in culture supernatants were assessed using bead array analysis with LEGENDplex Human Th Cytokine Panel (13-plex; BioLegend), performed according to the manufacturer's instructions and measured with an Aria II/III. Cytokine concentrations were calculated using the LEGENDplex v.8.0 data analysis software (BioLegend).

### Statistical analyses

Statistical analysis for each experiment is described in the figure legends. Descriptive data is shown as mean±s.e.m. if not indicated otherwise. Each *n* number represents an individual donor and a separate experiment. Differences between groups of data were analyzed via an unpaired, two-tailed Student's *t-*tests and two-way ANOVA. The software used for statistical analyses was GraphPad Prism 6.01 and *P-*values of less than 0.05 were considered significant.

## Supplementary Material

Supplementary information

Reviewer comments

## References

[DEV199781C1] Akgün, J., Prior, M. and Elbe-Bürger, A. (2014). Most T cells in human neonatal skin are not naive. *J. Clin. Exp. Dermatol. Res.* S2, 004.

[DEV199781C2] Armerding, D. and Kupper, T. S. (1999). Functional cutaneous lymphocyte antigen can be induced in essentially all peripheral blood T lymphocytes. *Int. Arch. Allergy Immunol* 119, 212-222. 10.1159/000024197 10436393

[DEV199781C3] Belle, M., Godefroy, D., Couly, G., Malone, S. A., Collier, F., Giacobini, P. and Chédotal, A. (2017). Tridimensional visualization and analysis of early human development. *Cell* 169, 161-173.e12. 10.1016/j.cell.2017.03.008 28340341

[DEV199781C4] Botting, R. A. and Haniffa, M. (2020). The developing immune network in human prenatal skin. *Immunology* 160, 149-156. 10.1111/imm.13192 32173857 PMC7218404

[DEV199781C5] Cahill, R. N., Kimpton, W. G., Washington, E. A., Holder, J. E. and Cunningham, C. P. (1999). The ontogeny of T cell recirculation during foetal life. *Semin. Immunol.* 11, 105-114. 10.1006/smim.1999.0166 10329497

[DEV199781C6] Carey, A. J., Hope, J. L., Mueller, Y. M., Fike, A. J., Kumova, O. K., Van Zessen, D. B. H., Steegers, E. A. P., Van Der Burg, M. and Katsikis, P. D. (2017). Public clonotypes and convergent recombination characterize the naïve CD8+ T-cell receptor repertoire of extremely preterm neonates. *Front. Immunol.* 8, 1859. 10.3389/fimmu.2017.01859 29312340 PMC5742125

[DEV199781C7] Carlson, B. (2012). *Human Embryology and Developmental Biology*, 5th edn. Elsevier.

[DEV199781C8] Clark, R. A., Chong, B. F., Mirchandani, N., Yamanaka, K.-I., Murphy, G. F., Dowgiert, R. K. and Kupper, T. S. (2006a). A novel method for the isolation of skin resident T cells from normal and diseased human skin. *J. Invest. Dermatol.* 126, 1059-1070. 10.1038/sj.jid.5700199 16484986

[DEV199781C9] Clark, R. A., Chong, B., Mirchandani, N., Brinster, N. K., Yamanaka, K.-, Dowgiert, R. K. and Kupper, T. S. (2006b). The vast majority of CLA+ T cells are resident in normal skin. *J. Immunol.* 176, 4431-4439. 10.4049/jimmunol.176.7.4431 16547281

[DEV199781C10] Clark, R. A., Watanabe, R., Teague, J. E., Schlapbach, C., Tawa, M. C., Adams, N., Dorosario, A. A., Chaney, K. S., Cutler, C. S., Leboeuf, N. R. et al. (2012). Skin effector memory T cells do not recirculate and provide immune protection in alemtuzumab-treated CTCL patients. *Sci. Transl. Med.* 4, 117ra7. 10.1126/scitranslmed.3003008 PMC3373186 22261031

[DEV199781C11] Colwell, A. S., Longaker, M. T. and Lorenz, H. P. (2003). Fetal wound healing. *Front. Biosci.* 8, s1240-s1248. 10.2741/1183 12957846

[DEV199781C12] Coolen, N. A., Schouten, K. C. W. M., Middelkoop, E. and Ulrich, M. M. W. (2010). Comparison between human fetal and adult skin. *Arch. Dermatol. Res.* 302, 47-55. 10.1007/s00403-009-0989-8 19701759 PMC2799629

[DEV199781C13] Cruz, M. S., Diamond, A., Russell, A. and Jameson, J. M. (2018). Human αβ and γδ T cells in skin immunity and disease. *Front. Immunol* 9, 1304. 10.3389/fimmu.2018.01304 29928283 PMC5997830

[DEV199781C14] Dhariwala, M. O., Karthikeyan, D., Vasquez, K. S., Farhat, S., Weckel, A., Taravati, K., Leitner, E. G., Clancy, S., Pauli, M., Piper, M. L. et al. (2020). Developing human skin contains lymphocytes demonstrating a memory signature. *Cell Reports Med* 1, 100132. 10.1016/j.xcrm.2020.100132 PMC7691438 33294857

[DEV199781C15] Di Nuzzo, S., Pavanello, P., Masotti, A., Giordano, G. and De, P. G. (2009). Densities, distribution and phenotypic expression of T cells in human fetal skin. *Arch. Dermatol. Res.* 301, 753-755. 10.1007/s00403-009-0943-9 19308434

[DEV199781C16] Ersch, J. and Stallmach, T. (1999). Assessing gestational age from histology of fetal skin: an autopsy study of 379 fetuses. *Obstet. Gynecol* 94, 753-757. 10546723 10.1016/s0029-7844(99)00379-8

[DEV199781C17] Han, X., Zhou, Z., Fei, L., Sun, H., Wang, R., Chen, Y., Chen, H., Wang, J., Tang, H., Ge, W. et al. (2020). Construction of a human cell landscape at single-cell level. *Nature* 581, 303-309. 10.1038/s41586-020-2157-4 32214235

[DEV199781C18] Ho, A. W. and Kupper, T. S. (2019). T cells and the skin: from protective immunity to inflammatory skin disorders. *Nat. Rev. Immunol.* 19, 490-502. 10.1038/s41577-019-0162-3 30992525

[DEV199781C19] Holbrook, K. A. and Odland, G. F. (1975). The fine structure of developing human epidermis: light, scanning, and transmission electron microscopy of the periderm. *J. Invest. Dermatol* 65, 16-38. 10.1111/1523-1747.ep12598029 168272

[DEV199781C20] Jung, D. and Alt, F. W. (2004). Unraveling V(D)J Recombination: insights into gene regulation. *Cell* 116, 299-311. 10.1016/S0092-8674(04)00039-X 14744439

[DEV199781C21] Mcgovern, N., Shin, A., Low, G., Low, D., Duan, K., Yao, L. J., Msallam, R., Low, I., Shadan, N. B., Sumatoh, H. R. et al. (2017). Human fetal dendritic cells promote prenatal T-cell immune suppression through arginase-2. *Nature* 546, 662-666. 10.1038/nature22795 28614294 PMC6588541

[DEV199781C22] Mielke, L. A., Jones, S. A., Raverdeau, M., Higgs, R., Stefanska, A., Groom, J. R., Misiak, A., Dungan, L. S., Sutton, C. E., Streubel, G. et al. (2013). Retinoic acid expression associates with enhanced IL-22 production by γδ T cells and innate lymphoid cells and attenuation of intestinal inflammation. *J. Exp. Med.* 210, 1117-1124. 10.1084/jem.20121588 23690441 PMC3674702

[DEV199781C23] Mildner, M., Prior, M., Gschwandtner, M., Schuster, C., Tschachler, E. and Elbe-Bürger, A. (2014). Epidermal CCL27 expression is regulated during skin development and keratinocyte differentiation. *J. Invest. Dermatol.* 134, 855-858. 10.1038/jid.2013.394 24037339

[DEV199781C24] Mold, J. E. and Mccune, J. M. (2012). Immunological tolerance during fetal development. From mouse to man. In *Advances in Immunology*, Vol. 115, pp. 73-111. Academic Press Inc. 22608256 10.1016/B978-0-12-394299-9.00003-5

[DEV199781C25] Morales, J., Homey, B., Vicari, A. P., Hudak, S., Oldham, E., Hedrick, J., Orozco, R., Copeland, N. G., Jenkins, N. A., Mcevoy, L. M. et al. (1999). CTACK, a skin-associated chemokine that preferentially attracts skin-homing memory T cells. *Proc. Natl. Acad. Sci. USA* 96, 14470-14475. 10.1073/pnas.96.25.14470 10588729 PMC24460

[DEV199781C26] Morath, A. and Schamel, W. W. (2020). αβ and γδ T cell receptors: Similar but different. *J. Leukoc. Biol.* 107, 1045-1055. 10.1002/JLB.2MR1219-233R 31994778

[DEV199781C27] Mukhopadhyay, M. (2021). Diving into the TCR repertoire. *Nat. Methods* 18, 30. 10.1038/s41592-020-01031-0 33408389

[DEV199781C28] Park, J. E., Jardine, L., Gottgens, B., Teichmann, S. A. and Haniffa, M. (2020). Prenatal development of human immunity. *Science* 368, 600-603. 10.1126/science.aaz9330 32381715 PMC7612900

[DEV199781C29] Popescu, D.-M., Botting, R. A., Stephenson, E., Green, K., Webb, S., Jardine, L., Calderbank, E. F., Polanski, K., Goh, I., Efremova, M. et al. (2019). Decoding human fetal liver haematopoiesis. *Nature* 574, 365-371. 10.1038/s41586-019-1652-y 31597962 PMC6861135

[DEV199781C30] Rechavi, E., Lev, A., Lee, Y. N., Simon, A. J., Yinon, Y., Lipitz, S., Amariglio, N., Weisz, B., Notarangelo, L. D. and Somech, R. (2015). Timely and spatially regulated maturation of B and T cell repertoire during human fetal development. *Sci. Transl. Med.* 7, 276ra25-276ra25. 10.1126/scitranslmed.aaa0072 25717098

[DEV199781C31] Reitermaier, R., Krausgruber, T., Fortelny, N., Ayub, T., Vieyra-Garcia, P. A., Kienzl, P., Wolf, P., Scharrer, A., Fiala, C., Kölz, M. et al. (2021). αβγδ T cells play a vital role in fetal human skin development and immunity. *J. Exp. Med* 218, e20201189. 10.1084/jem.20201189 33561194 PMC7876551

[DEV199781C32] Rojahn, T. B., Vorstandlechner, V., Krausgruber, T., Bauer, W. M., Alkon, N., Bangert, C., Thaler, F. M., Sadeghyar, F., Fortelny, N., Gernedl, V. et al. (2020). Single-cell transcriptomics combined with interstitial fluid proteomics defines cell type–specific immune regulation in atopic dermatitis. *J. Allergy Clin. Immunol*.146, 1056-1069. 10.1016/j.jaci.2020.03.041 32344053

[DEV199781C33] Rosati, E., Dowds, C. M., Liaskou, E., Henriksen, E. KK., Karlsen, T. H. and Franke, A. (2017). Overview of methodologies for T-cell receptor repertoire analysis. *BMC Biotechnol.* 17, 61. 10.1186/s12896-017-0379-9 28693542 PMC5504616

[DEV199781C34] Salimi, M., Subramaniam, S., Selvakumar, T., Wang, X., Zemenides, S., Johnson, D. and Ogg, G. (2016). Enhanced isolation of lymphoid cells from human skin. *Clin. Exp. Dermatol.* 41, 552-556. 10.1111/ced.12802 26805629 PMC4981906

[DEV199781C35] Sanchez Rodriguez, R., Pauli, M. L., Neuhaus, I. M., Yu, S. S., Arron, S. T., Harris, H. W., Yang, S. H.-Y., Anthony, B. A., Sverdrup, F. M., Krow-Lucal, E. et al. (2014). Memory regulatory T cells reside in human skin. *J. Clin. Invest.* 124, 1027-1036. 10.1172/JCI72932 24509084 PMC3934172

[DEV199781C36] Schlapbach, C., Gehad, A., Yang, C., Watanabe, R., Guenova, E., Teague, J. E., Campbell, L., Yawalkar, N., Kupper, T. S. and Clark, R. A. (2014). Human TH9 cells are skin-tropic and have autocrine and paracrine proinflammatory capacity. *Sci. Transl. Med.* 6, 219ra8. 10.1126/scitranslmed.3007828 PMC4102325 24431112

[DEV199781C37] Schuster, C., Vaculik, C., Fiala, C., Meindl, S., Brandt, O., Imhof, M., Stingl, G., Eppel, W. and Elbe-Bürger, A. (2009). HLA-DR+ leukocytes acquire CD1 antigens in embryonic and fetal human skin and contain functional antigen-presenting cells. *J. Exp. Med.* 206, 169-181. 10.1084/jem.20081747 19139172 PMC2626673

[DEV199781C38] Schuster, C., Vaculik, C., Prior, M., Fiala, C., Mildner, M., Eppel, W., Stingl, G. and Elbe-Bürger, A. (2012). Phenotypic characterization of leukocytes in prenatal human dermis. *J. Invest. Dermatol.* 132, 2581-2592. 10.1038/jid.2012.187 22718119 PMC3472563

[DEV199781C39] Schuster, C., Mildner, M., Mairhofer, M., Bauer, W., Fiala, C., Prior, M., Eppel, W., Kolbus, A., Tschachler, E., Stingl, G. et al. (2014). Human embryonic epidermis contains a diverse Langerhans cell precursor pool. *Development* 141, 807-815. 10.1242/dev.102699 24496618

[DEV199781C40] Schuster, C., Mildner, M., Botta, A., Nemec, L., Rogojanu, R., Beer, L., Fiala, C., Eppel, W., Bauer, W., Petzelbauer, P. et al. (2015). Development of blood and lymphatic endothelial cells in embryonic and fetal human skin. *Am. J. Pathol.* 185, 2563-2574. 10.1016/j.ajpath.2015.05.006 26188132

[DEV199781C41] Shevach, E. M. (2009). Mechanisms of Foxp3+ T regulatory cell-mediated suppression. *Immunity* 30, 636-645. 10.1016/j.immuni.2009.04.010 19464986

[DEV199781C42] Smith, L. T., Holbrook, K. A. and Madri, J. A. (1986). Collagen types I, III, and V in human embryonic and fetal skin. *Am. J. Anat.* 175, 507-521. 10.1002/aja.1001750409 3521252

[DEV199781C43] Watanabe, R., Gehad, A., Yang, C., Scott, L. L., Teague, J. E., Schlapbach, C., Elco, C. P., Huang, V., Matos, T. R., Kupper, T. S. et al. (2015). Human skin is protected by four functionally and phenotypically discrete populations of resident and recirculating memory T cells. *Sci. Transl. Med.* 7, 279ra39. 10.1126/scitranslmed.3010302 PMC4425193 25787765

[DEV199781C44] Zhang, X., Mozeleski, B., Lemoine, S., Deriaud, E., Lim, A., Zhivaki, D., Azria, E., Le Ray, C., Roguet, G., Launay, O. et al. (2014). CD4 T cells with effector memory phenotype and function develop in the sterile environment of the fetus. *Sci. Transl. Med.* 6, 238ra72. 10.1126/scitranslmed.3008748 24871133

[DEV199781C45] Zhang, Z., Xiong, D., Wang, X., Liu, H. and Wang, T. (2021). Mapping the functional landscape of T cell receptor repertoires by single-T cell transcriptomics. *Nat. Methods* 18, 92-99. 10.1038/s41592-020-01020-3 33408405 PMC7799492

[DEV199781C46] Zhao, Q., Eichten, A., Parveen, A., Adler, C., Huang, Y., Wang, W., Ding, Y., Adler, A., Nevins, T., Ni, M. et al. (2018). Single-cell transcriptome analyses reveal endothelial cell heterogeneity in tumors and changes following antiangiogenic treatment. *Cancer Res.* 78, 2370-2382. 10.1158/0008-5472.CAN-17-2728 29449267

[DEV199781C47] Zippelius, A., Bioley, G., Le Gal, F., Rufer, N., Brandes, M., Batard, P., De Smedt, M., Plum, J., Speiser, D. E., Cerottini, J.-C. et al. (2004). Human thymus exports naive CD8 T cells that can home to nonlymphoid tissues. *J. Immunol.* 172, 2773-2777. 10.4049/jimmunol.172.5.2773 14978076

