# Supplementary material for: The molecular and phenotypic makeup of fetal human skin T lymphocytes
Source: Development. 2021 Oct 26;149(8):dev199781. doi: 10.1242/dev.199781 (PMC8601710; doi:10.1242/dev.199781)
Supplement: Supplementary information [file develop-149-199781-s1.pdf]

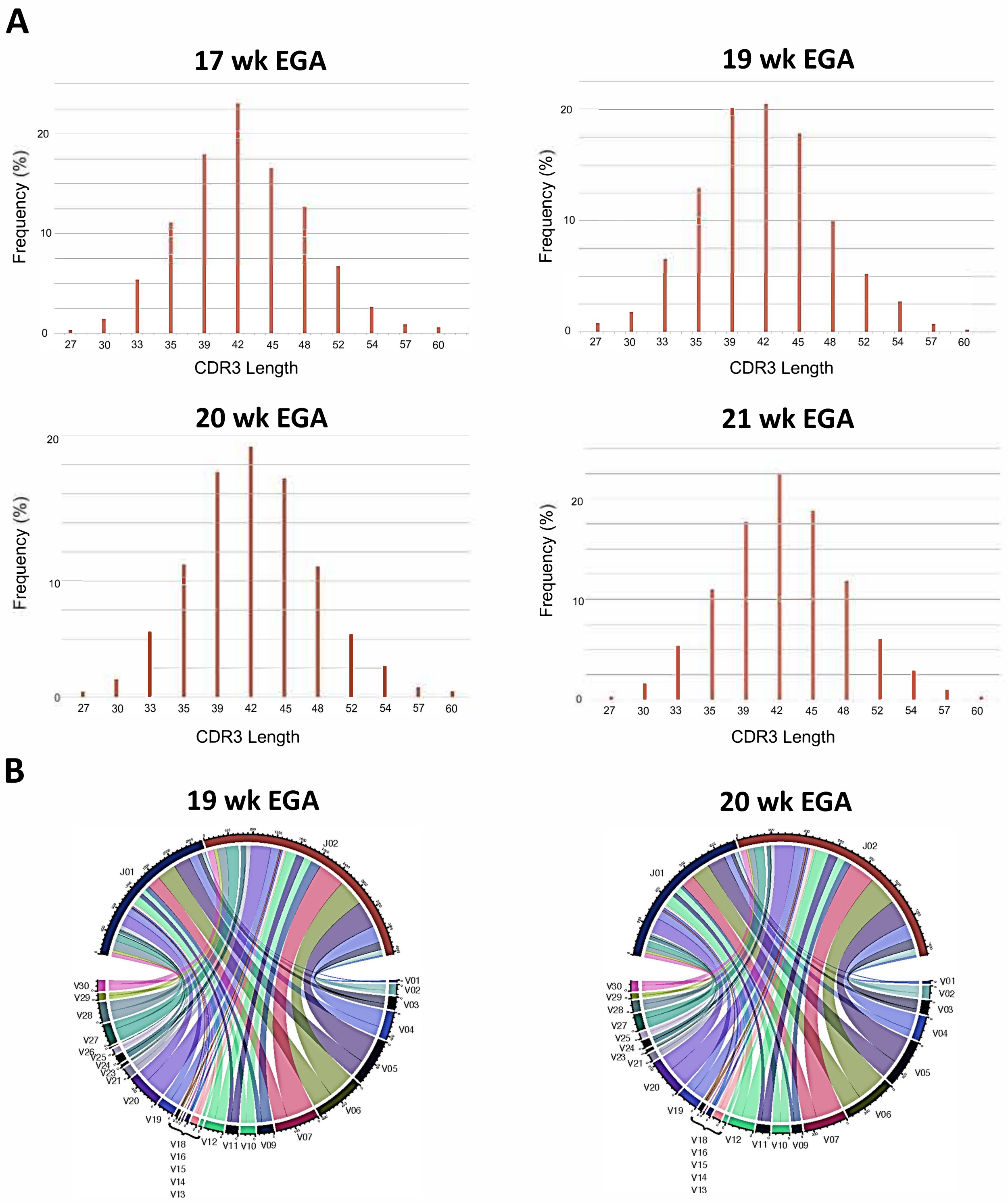

Fig. S1. High-throughput TCR Vβ CDR3 sequencing on fetal skin DNA. (A) Frequency distribution of the CDR3 length in fetal skin T cells (n=4). (B) Circos maps illustrating pairing frequencies of V- and J-segments from V(D)J-containing reads. Arrangements are presented in a clock-wise orientation from the top of the circle, in the order of J- and V-segments.

Development • Supplementary information

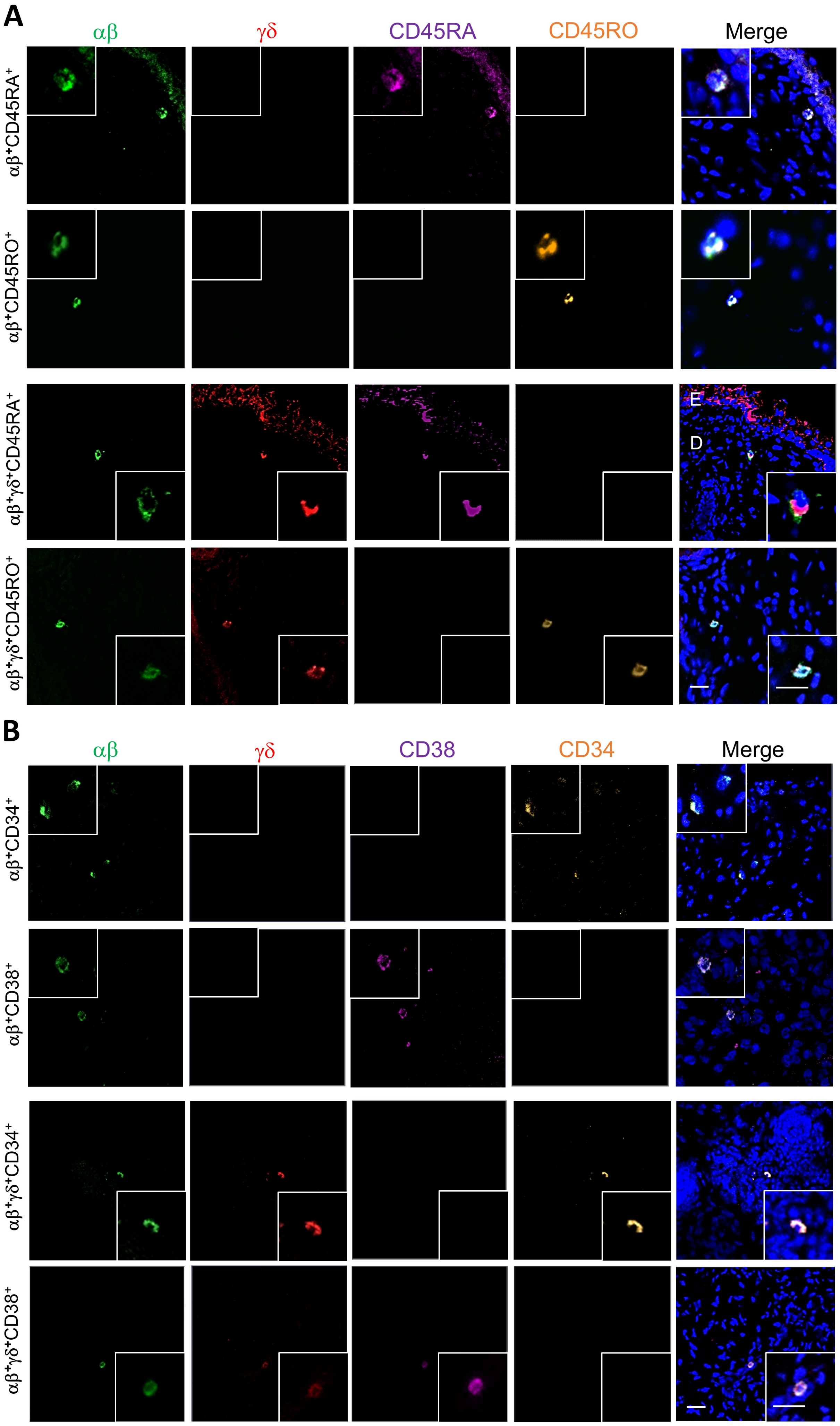

Development • Supplementary information

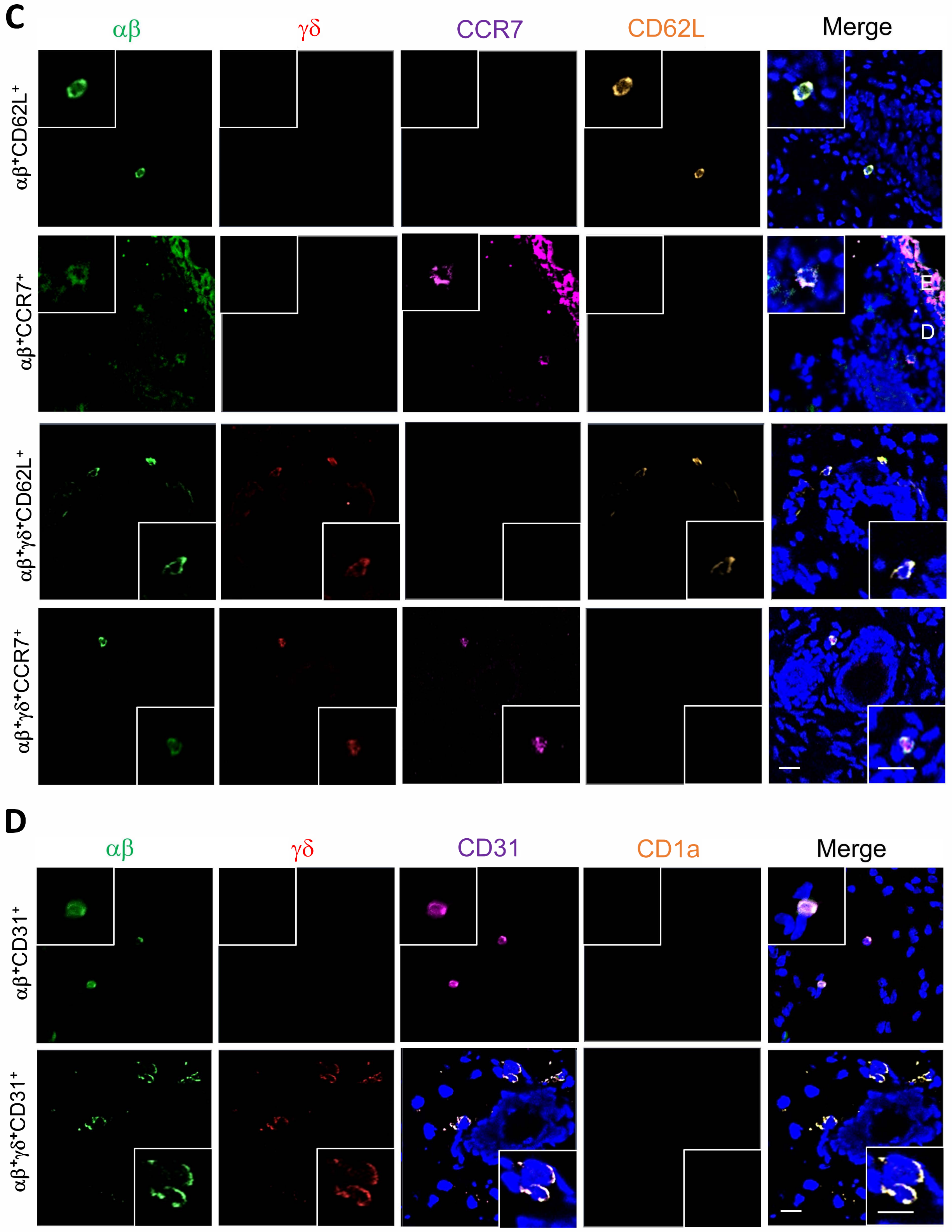

Fig. S2. *In situ* mapping of T cell subsets in fetal skin. (A-C) Immunofluorescence quadruple labelling and DAPI counterstaining for the markers indicated was performed on fetal skin cryostat sections and assessed by confocal laser microscopy. Images for each marker combination are representative from at least three different donors with similar results (17-22 weeks EGA; n=30). Scale bar: 20 µm. E=epidermis, D=dermis.

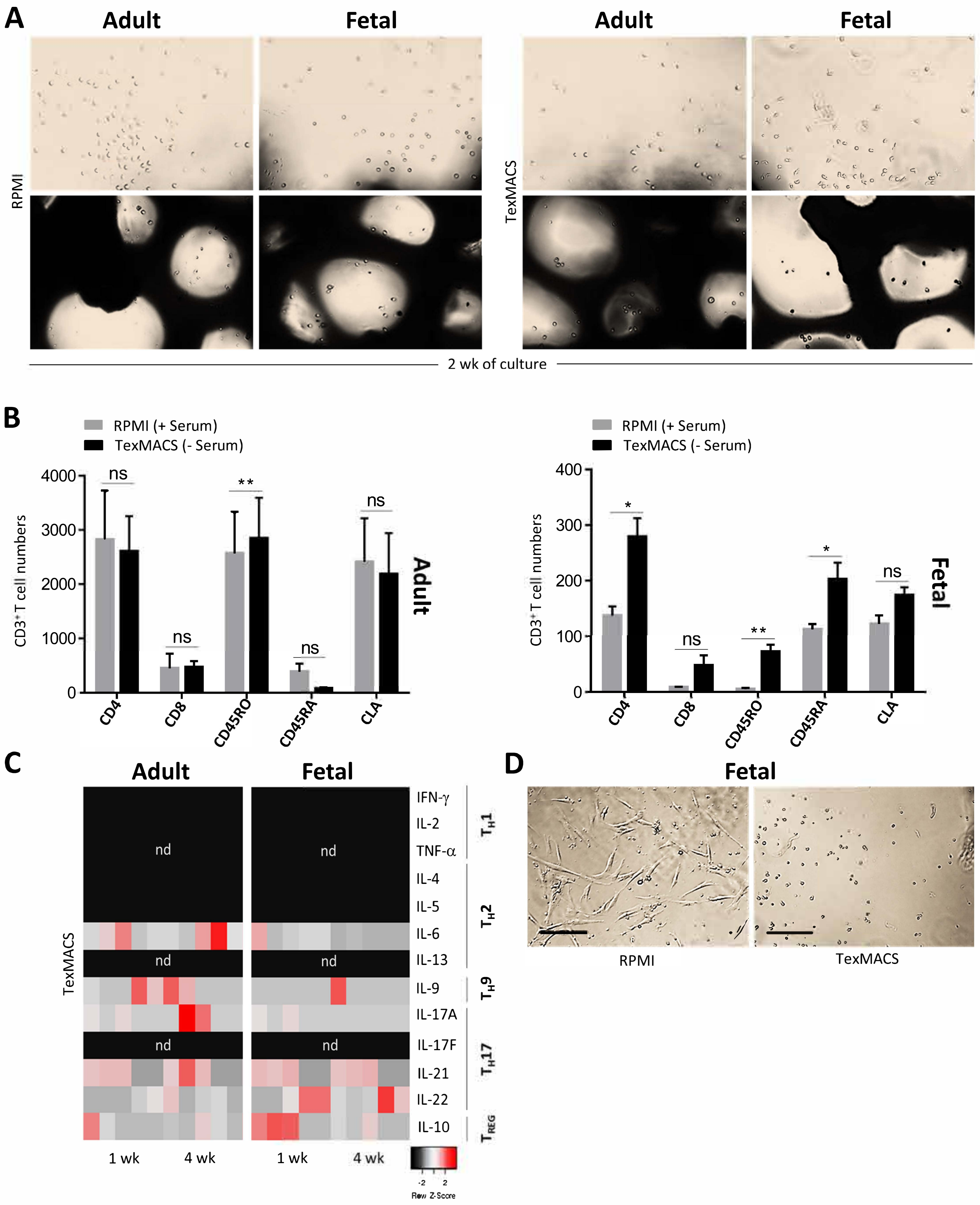

Fig. S3. Minimal expansion potential of T cells from fetal skin without cytokines. (A) Single T cells are visible upon culturing of fetal and, for comparison, adult skin specimens (n=8/age group) on collagen-treated grids in the absence of IL-2/15 in either RPMI or TexMACS medium after two weeks. Upper panels=nearby grid, lower panels=grid area. Scale bar: 200 µm. (B) Bar graphs (mean±SEM) showing numbers of expanded CD3^{+} T cells from fetal (n=5) and adult (n=8) donors using indicated culture conditions and flow cytometry analysis. Student`s *t*- test. *P≤0.01, **P≤0.009, ns=not significant. (C) Heat map of signature cytokines for each T cell subset identified in supernatants of fetal and adult skin specimen cultures (TexMACS) and measured with a cytometric bead assay at 1 and 4 weeks (n=5/age group). (D) Fibroblasts were regularly detected in cultures with RPMI and hardly in TexMACS medium. Shown is one representative experiment out of eight fetal donors. Scale bar: 200 µm.

Table S1. Antibodies

Antigen	Clone	Conjugation	Amount[a]	Isotype	Supplier
CD3	REA613<br>REA613<br>7D6	APC<br>PerCP-Vio700<br>PE-Texas Red	1µl<br>1µl<br>1µl	Recombinant human IgG1<br>Recombinant human IgG1<br>Mouse monoclonal IgG2a	Miltenyi Biotech<br>Miltenyi Biotech<br>Thermo Fisher Scientific
CD4	REA623	VioGreen	1µl	Recombinant human IgG1	Miltenyi Biotech
CD8	REA734<br>SK1	APC-H7<br>APC-H7	1µl<br>0.8µl	Recombinant human IgG1<br>Mouse monoclonal IgG1	Miltenyi Biotech<br>Becton Dickinson
αβ TCR	REA652<br>IP26	FITC<br>APC	1µl<br>1.1µl	Recombinant human IgG1<br>Mouse monoclonal IgG1	Miltenyi Biotech<br>Thermo Fisher Scientific
γδ TCR	REA591<br>B1.1	PE-Cy7<br>Per-CP eFluor 710	1µl<br>1.3µl	Recombinant human IgG1<br>Mouse monoclonal IgG1	Miltenyi Biotech<br>Thermo Fisher Scientific
Vδ1	REA173<br>REA173	APC<br>PE	1µl<br>1µl	Recombinant human IgG1<br>Recombinant human IgG1	Miltenyi Biotech<br>Miltenyi Biotech
Vδ2	REA771	PE	1µl	Recombinant human IgG1	Miltenyi Biotech
CCR2	REA264	APC	1µl	Recombinant human IgG1	Miltenyi Biotec
CCR4	D8SEE	APC	1µl	Mouse monoclonal IgG1	Thermo Fisher Scientific
CCR6	R6H1	Per-CP eFluor 710	1µl	Mouse monoclonal IgG1	Thermo Fisher Scientific
CCR7	REA546<br>3D12<br>150503	PerCp-Vio700<br>APC<br>Alexa Flour 594	1µl<br>0.9µl<br>1µl	Recombinant human IgG1<br>Mouse monoclonal IgG2a<br>Mouse monoclonal IgG2a	Miltenyi Biotec<br>Thermo Fisher Scientific<br>R&D Systems
CD1a	REA736<br>O10	APC-Vio770<br>Alexa Flour 594	1µl<br>1µl	Recombinant human IgG1<br>Mouse monoclonal IgG2a	Miltenyi Biotech<br>Miltenyi Biotech
CD25	REA570<br>MEM-181	APC<br>FITC	1.5µl<br>1.3µl	Recombinant human IgG1<br>Mouse monoclonal IgG1	Miltenyi Biotech<br>R&D Systems
CD27	323	FITC	0.9µl	Mouse monoclonal IgG1	Thermo Fisher Scientific
CD31	WM- 59<br>REA730	FITC<br>APC	1.3µl<br>1µl	Mouse monoclonal IgG1<br>Recombinant human IgG1	Thermo Fisher Scientific<br>Miltenyi Biotech
CD34	REA1164<br>756510	PerCP-Vio700<br>Alexa Flour 594	1µl<br>1.5µl	Recombinant human IgG1<br>Mouse monoclonal IgG2a	Miltenyi Biotech<br>R&D Systems
CD38	REA671<br>240742	PE-Vio770<br>Alexa Flour 647	1µl<br>1.5µl	Recombinant human IgG1<br>Mouse monoclonal IgG2a	Miltenyi Biotech<br>R&D Systems
CD39	eBioA1<br>A1	PE-Cy7<br>PE	1.8µl<br>1.5µl	Mouse monoclonal IgG1<br>Mouse monoclonal IgG1	Thermo Fisher Scientific<br>Mouse monoclonal IgG1
CD45RA	HI100<br>REA562<br>SPM568	PE<br>FITC<br>Alexa Flour 647	0.8µl<br>1µl<br>1µl	Mouse monoclonal IgG2a<br>recombinant human IgG1<br>Mouse monoclonal IgG2a	Becton Dickinson<br>Miltenyi Biotec<br>R&D Systems
CD45RO	UCHL1<br>SPM125	PerCP<br>Alexa Flour 594	1µl<br>1µl	Mouse monoclonal IgG2a<br>Mouse monoclonal IgG2a	Thermofischer<br>R&D Systems
CD56	TULY56	APC	1µl	Mouse monoclonal IgG1	Thermo Fisher Scientific
CD62L	DREG-56<br>IVA94	PE-Cyanine7<br>Alexa Flour 647	1.2µl<br>0.8µl	Mouse monoclonal IgG1<br>Mouse monoclonal IgG2a	Thermo Fisher Scientific<br>R&D Systems
CD69	FN50	Pe-Cy7	1µl	Mouse monoclonal IgG1	Thermo Fisher Scientific

Development • Supplementary information

CD73	AD2	FITC	1.3µl	Mouse monoclonal IgG1	Thermo Fisher Scientific
CD95	DX2	FITC	1.5µl	Mouse monoclonal IgG1	BioLegend
CD99	3B2/TA8	PE	1µl	Mouse monoclonal IgG2a	Thermo Fisher Scientific
CD103	Ber-ACT8	Per-CP eFluor 710	1µl	Mouse monoclonal IgG1	Thermo Fisher Scientific
CD152	14D3	Per-CP eFluor 710	1µl	Mouse monoclonal IgG2a	Thermo Fisher Scientific
CD159a (NKG2A)	REA110	PE	1µl	Recombinant human IgG1	Miltenyi Biotec
LAP	FNLAP	APC	1µl	Mouse monoclonal IgG1	Thermo Fisher Scientific
TIGIT	MBSA	Per-CP eFluor 710	1µl	Mouse monoclonal IgG1	Thermo Fisher Scientific
GARP	G14D9	PE-Cy7	1µl	Rat monoclonal IgG2a	Thermo Fisher Scientific
PLFZ	9E12	APC	1.5µl	Armenian hamster monoclonal IgG	Thermo Fisher Scientific
EOMES	WD1928	PE	1.5µl	Mouse monoclonal IgG1	Thermo Fisher Scientific
FoxP3	259/C7	PE	1.5µl	Mouse monoclonal IgG1	Becton Dickson
Ki67	20Raj1	FITC	1.5µl	Mouse monoclonal IgG1	Thermo Fisher Scientific
CLA	HECA-452	PE-Cy7	1µl	Rat monoclonal IgM	BioLegend
CXCL8	G265-8	PE	1.5µl	Mouse monoclonal IgG2b	Becton Dickinson
Collagen IV	Polyclonal	-	-	IgG	Abcam

[a]Per $1x10^5$/100µl

Development • Supplementary information
