# Supplementary material for: The molecular and phenotypic makeup of fetal human skin T lymphocytes
Source: Development. 2021 Oct 26;149(8):dev199781. doi: 10.1242/dev.199781 (PMC8601710; doi:10.1242/dev.199781)
Supplement: Reviewer comments [file develop-149-199781_review_history.pdf]

The molecular and phenotypic makeup of fetal human skin T lymphocytes

René Reitermaier, Tanya Ayub, Julia Staller, Philip Kienzl, Nikolaus Fortelny, Pablo Augusto Vieyra-Garcia, Christof Worda, Christian Fiala, Clement Staud, Wolfgang Eppel, Anke Scharrer, Thomas Krausgruber and Adelheid Elbe-Bürger
DOI: 10.1242/dev.199781

Editor: Paul Martin

Review timeline

Original submission:	5 May 2021
Editorial decision:	28 June 2021
First revision received:	14 July 2021
Accepted:	7 September 2021

Original submission

First decision letter

MS ID#: DEVELOP/2021/199781

MS TITLE: The molecular and phenotypic makeup of fetal human skin T lymphocytes

AUTHORS: Rene Reitermaier, Tanya Ayub, Julia Staller, Philip Kienzl, Nikolaus Fortelny, Pablo Augusto Vieyra-garcia, Christof Worda, Christian Fiala, Clement Staud, Wolfgang Eppel, Anke Scharrer, Thomas Krausgruber, and Adelheid Elbe-Burger

I have now received all the referees reports on the above manuscript, and have reached a decision. The referees' comments are appended below, or you can access them online: please go to BenchPress and click on the 'Manuscripts with Decisions' queue in the Author Area.

The overall evaluation is positive and we would like to publish a revised manuscript in Development, provided that the referees' comments can be satisfactorily addressed. Please attend to all of the reviewers' comments in your revised manuscript (including suggestions for additional experimental data) and detail them in your point-by-point response. If you do not agree with any of their criticisms or suggestions explain clearly why this is so.

We are aware that you may currently be unable to access the lab to undertake experimental revisions. If it would be helpful, we encourage you to contact us to discuss your revision in greater detail. Please send us a point-by-point response indicating where you are able to address concerns raised (either experimentally or by changes to the text) and where you will not be able to do so within the normal timeframe of a revision. We will then provide further guidance. Please also note that we are happy to extend revision timeframes as necessary.

Reviewer 1

Advance summary and potential significance to field

In this study, Reitermaier et al. describe the isolation expansion and characterization of T cells from human fetal skin. The authors compare T cell yields obtained using different methods and

© 2021. Published by The Company of Biologists under the terms of the Creative Commons Attribution License (https://creativecommons.org/licenses/by/4.0/).

using flow cytometry, immunofluorescence staining and RNA-seq the authors characterized the phenotype and molecular state of fetal skin T cell subsets.
Overall, the work provides new data on the characteristics of rare T cell subsets in human fetal skin. This study will allow the fields of skin development and skin immunity to progress and may provide useful insight for investigators working with small numbers of T cells resident in other solid tissues.

Comments for the author

This study would be enhanced with additional controls and certain technical aspects should be clarified to avoid confusion for the reader. Specifically:
(1) Can the authors comment on why the CD3 expression on fetal cells is so low in Figure 1 compared with Figures 3 and 4 and their previous JEM publication (ref 23)?

(2) Figure 2B shows distinct populations of αβ and γδ T cells (184 αβ and 216 γδ T cells according to the text). Of the 216 γδ T cells what proportion are DP αβγδT cells versus SP γδ T cells?

(3) A flow cytometry plot of αβ versus γδ should be included in Figure 4.

(4) Can the authors clarify their gating in Figure 4B? The figure legend indicates that CD3+ cells are shown in each plot, however it is not clear if additional gates may have been applied in some of the plots. For example very few cells are shown in the bottom left panel, yet there is an abundance of cells in each of the other panels from adult skin. Additionally, why is the proportion of CLA+ cells so variable (89.9%, 97.4% and 0.4% for the adult skin and 6.7%, 12.5% and 4.8% for the fetal skin)? Are these multicolor plots from a single donor or from 3 separate donors? This should be clarified in the text and figure legend. With so very few CLA+ cells in the fetal skin, the authors should show the isotype control stain which was used to define the CLA+ population.

(5) Also in Figure 4B, how does CD45RA and CD45RO expression in the fetal skin segregate with αβ versus γδ expression and CD4 versus CD8 expression?

(6) Figure 5 should include a comparison with adult skin. In addition, a better description of what is depicted in each panel should be provided in the figure legend. What does each color represent in Figure 5B? Are these the same images as the merged panels in Supplementary Figure 2, although some seem to have the inset panel in a different location?

(7) It would be helpful to have a more detailed description of the ATD+DK method in the materials and methods section.

(8) Figure 1A is missing a y-axis label.

(9) Some details on statistics are missing (Figures 3B and 6F).

Reviewer 2

Advance summary and potential significance to field

The findings from this study will be a useful contribution to the community.
The authors describe human fetal T cells using several orthogonal approaches as well as a technique to expand fetal T cells ex vivo.

I have several points requiring clarification and additional experimental support:

1. The scRNA-seq data is under utilised. Despite the low numbers Figure 3 does not show any attempt to sub cluster the T cells to look for T cell subsets that may be present. This ought to be done.

2. Please detail how many CD3- and CD3+ cells were loaded for 10x and if the vast majority of T cells were recovered from the CD3+ fraction.

© 2021. Published by The Company of Biologists under the terms of the Creative Commons Attribution License (https://creativecommons.org/licenses/by/4.0/).

3. Why are some erythrocytes (pink) clustering with NK cells (grey) in Fig 2B?

4. Is Figure 4A from bulk RNA-seq or pseudobulb analysis of scRNA-seq data?

5. Why are SP and DP T cells expressing CD34? They shouldn't be expressing progenitor markers which are lost once lymphoid progenitors differentiate into T cells.

6. It would be good to see flow cytomtery analysis of the fetal T cells propagated ex vivo to compare with freshly isolated T cells? I think the comment that they are similar to freshly isolated cells should be removed or caveated as the comparisons were not comprehensive.

7. The discussion could be more succinct.

Comments for the author

The findings from this study will be a useful contribution to the community. I have several points requiring clarification and additional experimental support:
1. The scRNA-seq data is under utilised. Despite the low numbers Figure 3 does not show any attempt to sub cluster the T cells to look for T cell subsets that may be present. This ought to be done.

2. Please detail how many CD3- and CD3+ cells were loaded for 10x and if the vast majority of T cells were recovered from the CD3+ fraction.

3. Why are some erythrocytes (pink) clustering with NK cells (grey) in Fig 2B?

4. Is Figure 4A from bulk RNA-seq or pseudobulb analysis of scRNA-seq data?

5. Why are SP and DP T cells expressing CD34? They shouldn't be expressing progenitor markers which are lost once lymphoid progenitors differentiate into T cells.

6. It would be good to see flow cytomtery analysis of the fetal T cells propagated ex vivo to compare with freshly isolated T cells? I think the comment that they are similar to freshly isolated cells should be removed or caveated as the comparisons were not comprehensive.

7. The discussion could be more succinct.

First revision

Author response to reviewers' comments

Author´s Response

Dear Dr. Martin,
Thank you for having considered our manuscript "The molecular and phenotypic makeup of fetal human skin T lymphocytes" (DEVELOP/2021/199781) for publication in Development. We appreciate the time and effort that you and the reviewer dedicated to providing feedback on our manuscript and are grateful for the insightful comments and valuable improvements to our paper.

Please find enclosed our detailed point-by-point reply to all the questions and suggestions raised by the reviewers. Our changes/additions in the manuscript are highlighted in yellow and all page numbers refers to the revised manuscript. We do hope for a favorable decision.

Sincerely,
Adelheid Elbe-Bürger

© 2021. Published by The Company of Biologists under the terms of the Creative Commons Attribution License (https://creativecommons.org/licenses/by/4.0/).

Authors responses to the general remarks of the reviewers. We thank the reviewers for their interest and their positive view of our work and for their insightful and helpful comments, questions and suggestions.

Reviewer #1:

Comment 1
Can the authors comment on why the CD3 expression on fetal cells is so low in Figure 1 compared with Figures 3 and 4 and their previous JEM publication (ref 23)?

Response to comment 1
We apologize for not having described these details in the manuscript and thank the reviewer for bringing this important and obviously misleading point up. To be able to compare the cell yields using different isolation techniques, it was mandatory to use the same amount of skin tissue (3 biopsies à ∅ 4 mm) from the identical donor. For analyses such as those in Figures 3 and 4 (this manuscript) and in our previous publication (doi: 10.1084/jem.20201189), we indeed pooled single cell suspensions from several punch biopsies from one donor to be able to analyze T cells in more depths. Further, biological variability among donors (e.g. different age) also play an important role as reported (doi: 10.1084/jem.20201189). We have added this information in the Method (page 3) and Result (page 6) sections of the revised manuscript (highlighted in yellow).

Comment 2
Figure 2B shows distinct populations of αβ and γδ T cells (184 αβ and 216 γδ T cells according to the text). Of the 216 γδ T cells what proportion are DP αβγδ T cells versus SP γδ T cells?
Response to comment 2 Of the 216 γδ T cells we had 100 DP αβγδ, and 116 SP γδ) T cells, while in the αβ T cell cluster no DP αβγδ cells were identified. We have now added this information in the Results of the revised manuscript (page 7) (highlighted in yellow).

Comment 3
A flow cytometry plot of αβ versus γδ should be included in Figure 4.

Response to comment 3
As requested we have included a representative contour plot (revised Figure 4B) and further reorganized Figure 4 also according to comment 4 from reviewer 1 and added this information in the Results (pages 7, 8) and respective figure legend (highlighted in yellow).

Comment 4
Can the authors clarify their gating in Figure 4B?
The figure legend indicates that CD3+ cells are shown in each plot, however it is not clear if additional gates may have been applied in some of the plots. For example, very few cells are shown in the bottom left panel, yet there is an abundance of cells in each of the other panels from adult skin. Additionally, why is the proportion of CLA+ cells so variable (89.9%, 97.4% and 0.4% for the adult skin and 6.7%, 12.5% and 4.8% for the fetal skin)? Are these multicolor plots from a single donor or from 3 separate donors? This should be clarified in the text and figure legend.
With so very few CLA+ cells in the fetal skin, the authors should show the isotype control stain which was used to define the CLA+ population.

Response to comment 4
-- The following gating strategy was applied in the revised Figure 4B-E: upon doublet and dead cells exclusion, αβ/γδ/CD4/CD8/CD45RO/RA expression was analyzed on adult- and fetal skin-derived CD3+ T cells and has been explained in the original Methods section (page 4).
-- CLA expression was investigated on one hand on total CD3+ T cells and on the other hand on CD3+CD45RO and CD3+CD45RA T cell subsets obtained from different donors.
-- As the reviewer rightly says, there are overall “seemingly” discrepancies with the CLA expression. The variability of CLA+ cells in Figure 4D and E can be explained by biological variables between the different donors, even though both adult donors were of similar age [36 years (D) vs. 38 years (E)] and, moreover, fetal donors were even of the same age (18 weeks EGA). This information has now been included in the revised version of the manuscript (page 8).
-- As requested, we provide an isotype control for the CLA staining (revised Figure 4D)

© 2021. Published by The Company of Biologists under the terms of the Creative Commons Attribution License (https://creativecommons.org/licenses/by/4.0/).

Comment 5
Also in Figure 4B, how does CD45RA and CD45RO expression in the fetal skin segregate with αß versus γδ expression and CD4 versus CD8 expression?

Response to comment 5
This is an excellent argument and has been addressed in our previous publication in detail (Figure 3K and L) (doi: 10.1084/jem.20201189). Unfortunately, due to the very low numbers of fetal SP γδ T cells further analyses on this cell type could not be performed.

Comment 6

Figure 5 should include a comparison with adult skin.
In addition, a better description of what is depicted in each panel should be provided in the figure legend. What does each color represent in Figure 5B?
Are these the same images as the merged panels in Supplementary Figure 2, although some seem to have the inset panel in a different location?

Response to comment 6
-- We think that the enclosure of adult skin is unreasonable as DP αßγδ cells are not present in adult skin and the focus of our paper is the characterization of fetal human skin T cell subsets. Moreover, staining profiles of healthy adult human skin with markers used in Figure 5 were published several times from different groups already.
-- Indeed, because of space issues, we have provided merged images for all antibody combinations in Figure 5B and have shown split images in Supplementary Figure 2A-D.
-- The reviewer is correct indicating that the location of some insets in the Supplementary Figure 2 are different compared to the main Figure 5B. Our intention was to provide a better visibility, but we agree that this might be confusing. Accordingly, we have rearranged the insets in the Supplementary Figure 2 and now correspond completely to Figure 5B.

Comment 7
It would be helpful to have a more detailed description of the ATD+DK method in the materials and methods section.

Response to comment 7
We followed the manufacturer´s description as initially already described in the Methods section and have added minor details in the revised manuscript (page 3) (highlighted in yellow).

Comment 8
Figure 1A is missing a y-axis label.
Response to comment 8
We thank the reviewer for the attention to details and have added the y-axis label in Figure 1A.

Comment 9
Some details on statistics are missing (Figures 3B and 6F).
Response to comment 9
We apologize for this oversight and have added the missing statistics in the respective figure legends of the revised manuscript (highlighted in yellow).

Reviewer #2:
Comment 1
The scRNA-seq data is under utilised. Despite the low numbers Figure 3 does not show any attempt to sub cluster the T cells to look for T cell subsets that may be present. This ought to be done.

Response to comment 1
In Figure 3 we describe the distribution of the TCR Vß repertoire in fetal skin T cells using flow cytometry (Figure 3B) and high-throughput sequencing (Figure 3D and E). We assume that the reviewer was referring to scRNA-seq data provided in Figure 2. A more detailed scRNA-seq analysis of T cell subsets is provided in our previous publication (doi: 10.1084/jem.20201189). A comprehensive analysis is currently in progress and adding some of these data, which are planned for a new manuscript with another topic, would be certainly beyond the scope of this paper.

© 2021. Published by The Company of Biologists under the terms of the Creative Commons Attribution License (https://creativecommons.org/licenses/by/4.0/).

Comment 2
Please detail how many CD3- and CD3+ cells were loaded for 10x and if the vast majority of T cells were recovered from the CD3+ fraction.

Response to comment 2
The reviewer might have overseen that we have described this in detail already on page 4 in the Methods section in the originally submitted version of the manuscript (“Viable, sorted CD3+ T cells (up to 5.000 cells/fetus) were mixed with sort-purified total skin cells (ratio up to 1:1) to get a more complete resolution of human skin development.”). We do hope that this is what the reviewer had in mind? If not, we will certainly add relevant information if the reviewer specifies it in more detail.

Comment 3
Why are some erythrocytes (pink) clustering with NK cells (grey) in Fig 2B?

Response to comment 3
Dimensionality reduction methods, such as t-SNE and UMAP, as well as clustering methods, such as graph-based clustering, are affected by noise in the data. As a result, there can be disagreement between the clusters visually discerned from the t-SNE and those identified by clustering (which ended up as our cell-type labels). Since we focused on detailed analysis of T cells here, and T cells were readily distinguishable from other cell types, we did not further optimize the above unsupervised analyses.

Comment 4
Is Figure 4A from bulk RNA-seq or pseudo bulb analysis of scRNA-seq data?

Response to comment 4
We apologize for not having specified this in the Results and the Figure legend and are grateful that the reviewer noted this. Data in Figure 4A result from comprehensive flow cytometry analyses. We have now added this information in the Result section (page 7) and the respective figure legend (page 16) of the revised manuscript (highlighted in yellow).

Comment 5
Why are SP and DP T cells expressing CD34? They shouldn't be expressing progenitor markers which are lost once lymphoid progenitors differentiate into T cells.

Response to comment 5
We agree with the reviewer and are puzzled as well. Based on the expression of CD34 and the absence of DP T cells in the thymus, we suppose that DP and some SP T cells do not undergo the “classical” maturation steps in the thymus and hypothesize an extrathymic development of these cells in the skin, which is an exciting but not new idea and has to be further investigated.

Comment 6
It would be good to see flow cytometry analysis of the fetal T cells propagated ex vivo to compare with freshly isolated T cells? I think the comment that they are similar to freshly isolated cells should be removed or caveated as the comparisons were not comprehensive.

Response to comment 6
We agree with the reviewer that this is absolutely required. We have addressed this in depths in our previous publication, showing a comparative flow cytometry analysis of freshly isolated versus expanded fetal skin T cells using cardinal T cell markers (Figure 5D in doi: 10.1084/jem.20201189).

Comment 7
The discussion could be more succinct.
Response to comment 7
As our manuscript does not exceed the permitted word count, we would like to keep the length of the discussion and not disturb the current flow.

© 2021. Published by The Company of Biologists under the terms of the Creative Commons Attribution License (https://creativecommons.org/licenses/by/4.0/).

Second decision letter

MS ID#: DEVELOP/2021/199781

MS TITLE: The molecular and phenotypic makeup of fetal human skin T lymphocytes

AUTHORS: Rene Reitermaier, Tanya Ayub, Julia Staller, Philip Kienzl, Nikolaus Fortelny, Pablo Augusto Vieyra-garcia, Christof Worda, Christian Fiala, Clement Staud, Wolfgang Eppel, Anke Scharrer, Thomas Krausgruber, and Adelheid Elbe-Burger
ARTICLE TYPE: Techniques and Resources Article

I am happy to tell you that your manuscript has been accepted for publication in Development, pending our standard ethics checks.

Reviewer 1

Advance summary and potential significance to field

In this study, Reitermaier et al. describe a new method for isolating and expanding T cells from human fetal skin. The authors compare T cell yields obtained using their new method with more conventional isolation methods.
Using flow cytometry, immunofluorescence staining and RNA-seq the authors characterized the phenotype and molecular state of fetal skin T cell subsets.
Overall, the work provides a new method to isolate and expand rare T cell populations. This study will allow the fields of skin development and skin immunity to progress and may provide useful insight for investigators working with small numbers of T cells resident in other solid tissues.

Comments for the author

The authors have adequately addressed the majority of reviewer concerns.

© 2021. Published by The Company of Biologists under the terms of the Creative Commons Attribution License (https://creativecommons.org/licenses/by/4.0/).
